# No Reliable Evidence for a Neanderthal-Châtelperronian Association at La Roche-à-Pierrot, Saint-Césaire

**DOI:** 10.1038/s41598-018-33084-9

**Published:** 2018-10-11

**Authors:** Brad Gravina, François Bachellerie, Solène Caux, Emmanuel Discamps, Jean-Philippe Faivre, Aline Galland, Alexandre Michel, Nicolas Teyssandier, Jean-Guillaume Bordes

**Affiliations:** 1 UMR-5199, PACEA, Université de Bordeaux, Bâtiment B8, Allée Geoffroy Saint Hilaire, CS 50023, 33615 PESSAC CEDEX, France; 2 Archéologie Alsace 11 Rue Jean-François Champollion, 67600 Sélestat, France; 3 Centre de Recherche Français, Jerusalem, Israel; 4 UMR-5608, TRACES, Université Toulouse – Jean Jaurès, Maison de la Recherche, 5 allée Antonio Machado, 31058 Toulouse Cedex 9, France; 5 Service de l’Archéologie, Département de la Dordogne, Hôtel du Département, CS11200, 24019 Périgueux, France

## Abstract

The demise of Neanderthals and their interaction with dispersing anatomically modern human populations remain some of the most contentious issues in palaeoanthropology. The Châtelperronian, now generally recognized as the first genuine Upper Palaeolithic industry in Western Europe and commonly attributed to the Neanderthals, plays a pivotal role in these debates. The Neanderthal authorship of this techno-complex is based on reported associations of Neanderthal skeletal material with Châtelperronian assemblages at only two sites, La Roche-à-Pierrot (Saint-Césaire) and the Grotte du Renne (Arcy-sur-Cure). The reliability of such an association has, however, been the subject of heated controversy. Here we present a detailed taphonomic, spatial and typo-technological reassessment of the level (EJOP sup) containing the Neanderthal skeletal material at Saint-Césaire. Our assessment of a new larger sample of lithic artifacts, combined with a systematic refitting program and spatial projections of diagnostic artifacts, produced no reliable evidence for a Neanderthal-Châtelperronian association at the site. These results significantly impact current models concerning the Middle-to-Upper Palaeolithic transition in Western Europe and force a critical reappraisal of who exactly were the makers of the Châtelperronian.

## Introduction

Documenting the emergence of traits commonly considered as proxies for ‘behavioural modernity’ remains one of the most debated research challenges in Palaeolithic archaeology. Over the last two decades, new analyses in Africa have pushed back the earliest occurrences of such evidence^1–12^, which were previously thought to be coincident with the Middle-to-Upper Palaeolithic transition in Western Europe. Despite intense dispute as to whether such evidence is unique to anatomically modern human populations, it now appears clear that Middle Palaeolithic Neanderthal populations across their known range also made and used bone tools^13,14^, sourced and processed pigments^15–18^, potentially buried their dead^19–25^, and probably possessed elements of personal ornamentation^26–29^.

The Châtelperronian, commonly assumed to be one of the final cultural manifestations of Neanderthals in Western Europe, is pivotal to this debate. Its unique features combining blade and bladelet technologies alongside personal ornaments, bone tools and considerable pigment use has simultaneously been interpreted as support either for the acculturation of final Neanderthal populations by dispersing anatomically modern human groups^30–36^ or the independent emergence of cultural innovations amongst the former^37–40^. The Neanderthal authorship of the Châtelperronian is, however, based on reported direct associations of Neanderthal skeletal remains with Châtelperronian cultural material at only two sites — scattered teeth, a temporal bone and bone fragments from the lowermost Châtelperronian levels of the Grotte du Renne at Arcy-sur-Cure^32,41,42^ and a partial Neanderthal skeleton in a level (EJOP sup) attributed to the Châtelperronian at La Roche-à-Pierrot, Saint-Césaire^43–45^. However, important reservations persist concerning the reliability of the Neanderthal-Châtelperronian association at both sites^46–50^.

Despite the paramount importance of Saint-Césaire for almost all models for the Middle-to-Upper Paleolithic transition in Western Europe, the limited amount of published information concerning the site’s lithic assemblages^51–54^ makes it difficult to evaluate the reliability of the reported stratigraphic association of Neanderthal skeletal elements with diagnostic Châtelperronian artefacts. Here we report a detailed spatial, taphonomic and techno-typological reassessment of the level containing the partial Neanderthal skeleton designed to thoroughly re-evaluate its cultural attribution and stratigraphic integrity and ultimately discuss the context of the Neanderthal skeletal remains.

## La Roche-à-Pierrot, Saint-Césaire

Located in the Charente-Maritime department of southwestern France and excavated by F. Lévêque for 12 uninterrupted seasons between 1976 and 1987, the site of La-Roche-à-Pierrot, Saint-Césaire lies at the base of an Upper Turonian limestone cliff (see Supplementary Information for details of site history, Supplementary Figs 1 and 2). According to the original excavators, this small collapsed rockshelter documents the complete Mousterian-Châtelperronian-Aurignacian cultural sequence^55^.

Two levels reported as containing Châtelperronian cultural material were identified during excavations; an upper pale yellow-orange level (EJOP sup) with abundant angular blocks underlain by a level of the same colour (EJOP inf) but with fewer blocks and a more clayey matrix, whose base (EJOP inf base) contained numerous large blocks. It is also important to note that prior to the distinction of these subdivisions the material recovered throughout the pale yellow-orange level was noted simply as EJOP, including at the time the Neanderthal remains were discovered.

While Lévêque’s presentation of the lithic material from EJOP sup concentrated solely on the 305 retouched tools recovered from all squares where this level was identified (32 sq. metres), a more recent analysis^53,54^ focused on a larger sample of 2594 flakes greater than 3 cm, 193 cores and 201 tools combining all four stratigraphic designations (EJOP, EJOP inf base, EJOP inf and EJOP sup). Importantly, with the help of Lévêque and reference to information in the field notebooks (e.g., artefact altitudes, sedimentological observations), an unspecified amount of material from the undifferentiated EJOP level was reassigned to EJOP inf or sup^53,54^. This reanalysis resulted in the reaffirmation of Lévêque’s original attribution of EJOP sup to the Châtelperronian and at the same time noted that: (1) Mousterian and Châtelperronian artefacts do not significantly differ in terms of surface alterations, (2) EJOP inf is more appropriately defined as Mousterian, (3) EJOP sup nevertheless still portrays a considerable Mousterian ‘aspect’, and (4) that overall it remains difficult to discern whether the particular composition of the Châtelperronian of Saint-Césaire is cultural or the product of post-depositional mixing.

Considerable research concerning Châtelperronian lithic technology has since demonstrated this techno-complex to consist of a fully Upper Paleolithic blade/bladelet technology^50,55–61^ and that the presence of typical Mousterian artefacts can be attributed to post-depositional admixture from underlying deposits^57,62^. Additionally, in taphonomically tested contexts (e.g. Quincay, Canaule II), Mousterian tool types were found to be manufactured uniquely on by-products from systematic blade production and not blanks issuing from any of the now well-documented Mousterian flake production systems^59,63,64^. These recent advances in the techno-typological definition of the Châtelperronian form the basis of our reanalysis of level EJOP sup, which aimed to test both the cultural homogeneity of EJOP sup and whether there exists any reliable evidence for a Neanderthal-Châtelperronian association at Saint-Césaire.

## Materials and Methods

To properly assess its cultural homogeneity, we combined vertical and horizontal projections of piece-plotted artefacts in order to reconstruct the largest reliable assemblage of lithic artefacts from EJOP sup. This reconstituted assemblage was then the focus of a detailed typo-technological analysis designed to isolate potentially different chrono-cultural components and explore their spatial relationship to the Neanderthal remains. Finally, we integrated a systematic conjoining program with an evaluation of lithic surface alterations, including a targeted analysis using confocal microscopy, to investigate the potential reworking and redistribution of the EJOP sup lithic material. Note that nearly 1000 person-hours were necessary to recondition the remaining unwashed, unmarked, and un-inventoried lithic objects (approximately 90% of the total lithic material) from the site’s Upper Palaeolithic levels, the Châtelperronian included.

### Reconstituting the EJOP sup lithic assemblage

**Figure 1:**
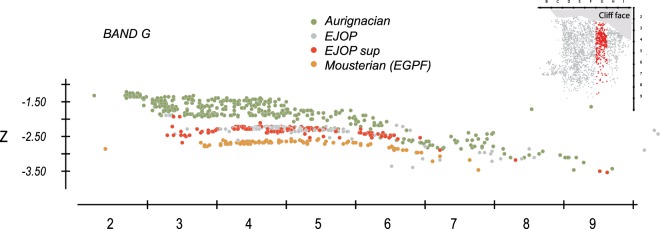
Projection of all lithic material recovered from band G during Lévêque’s excavations. Note that from line 6 onwards the heavily sloped deposits mix material assigned by Lévêque to the Mousterian, Châtelperronian and Aurignacian.

Up until now, the absence of spatial projections of the archaeological material substantially hindered our ability to evaluate both the Saint-Césaire stratigraphy and the context of the Neanderthal skeletal material. Fortunately, Lévêque marked the coordinates directly on each piece-plotted artefact (Supplementary Information, Supplementary Fig. S4). However, only approximately 15% of over 42,000 lithic objects greater than 1.5 cm from the various stratigraphic designations of EJOP (i.e. base, inf, sup, or undifferentiated) were piece-plotted during excavations. Our spatial analysis of all of the piece-plotted objects demonstrates that, beginning from line 6, the Upper Palaeolithic levels, including those attributed to the Châtelperronian, slope significantly south-east – north-west, where they become compressed and intercalated (Fig. 1). This inevitably led to substantial mixing and significant difficulties in the stratigraphic attribution of the material during excavations in the down slope area of the site (i.e. lines 6 to 9), a problem readily admitted by the original excavators, and further complicated by the fact that the site was not systematically excavated horizontally following the levels.

**Figure 2:**
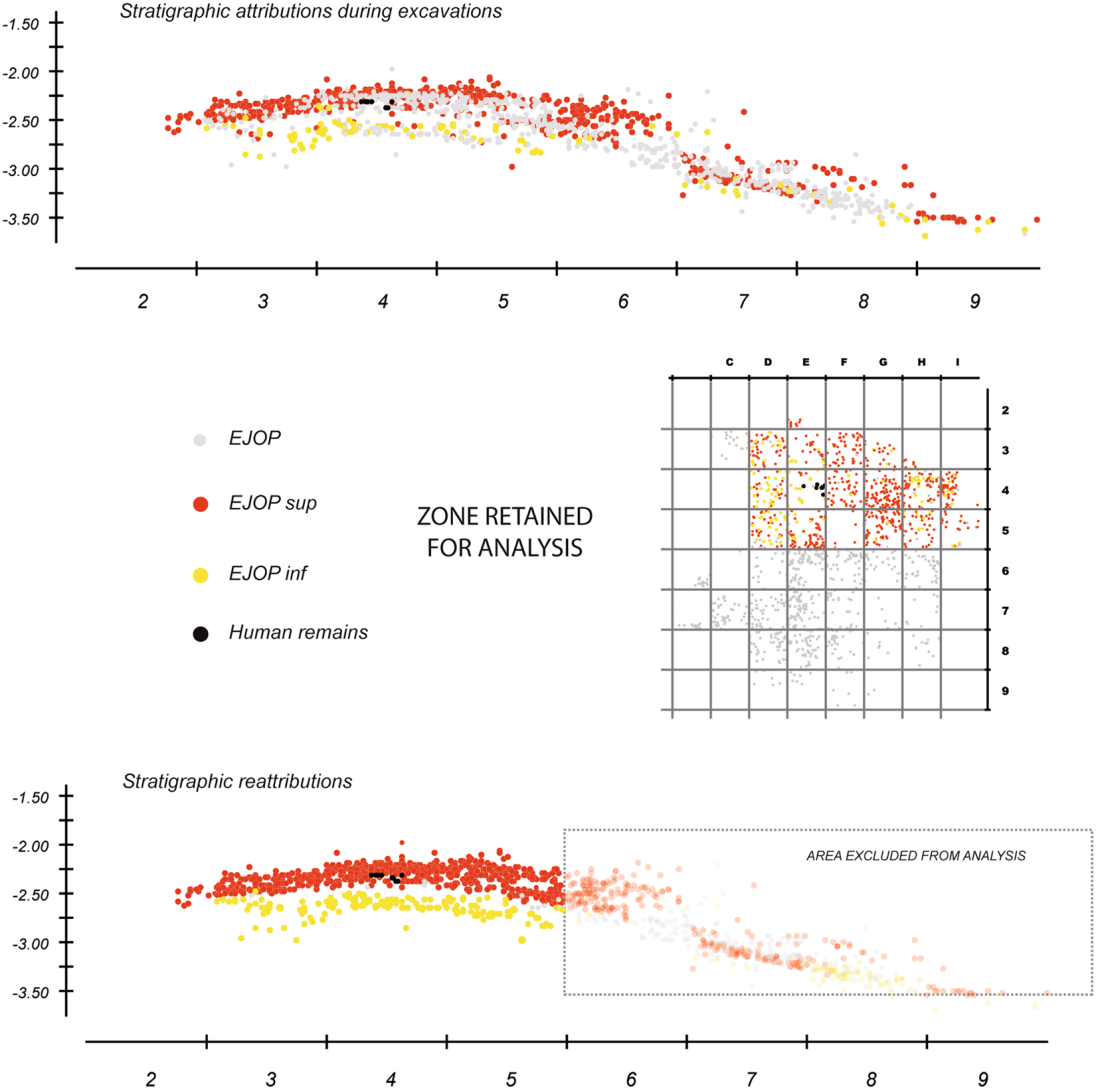
Projection of lithic material recovered from the pale-orange yellow level (EJOP, EJOP inf, EJOP sup) during Lévêque’s excavations (above) compared with our reattributions (below).

On the other hand, in lines 2 to 5, which include the Neanderthal skeletal material, the most consequential archaeological levels are relatively horizontal, sloping only slightly towards the cliff and, to a lesser extent, toward the south-west (Fig. 2). In order to best avoid any artificial mixing of material from different levels and to have the most representative sample possible, we concentrated our analysis on the material recovered from this 17 m² area, approximately the same as previously studied^53,54^.

Separating the two major EJOP sub-levels (EJOP inf and sup) during excavations proved difficult, which is equally evident from our projections of the lithic material, with nearly 50% of the piece-plotted material attributed to an undifferentiated EJOP (n = 9211). Our fine-grained stratigraphic and spatial analyses using projections of the piece-plotted material allowed a much larger portion of the undifferentiated EJOP assemblage to be re-attributed to one of the two sub-levels (EJOP inf or sup) with greater precision and confidence than was previously possible, thus augmenting the piece-plotted EJOP sup sample by 40% (Fig. 2) compared to prior analyses. These reattributions equally allowed us to assign or exclude non-piece plotted material in the retained zone to EJOP sup based on the newly defined vertical limits of this level in each 50 cm by 50 cm sub-square (Supplementary Information, Supplementary Fig. S3). Here it is important to note that the lower limits of each spit reassigned to EJOP sup, as well as those originally attributed by the excavators to this unit, fall within the sterile band and above any potential stratigraphic disconformity with the underlying Mousterian (see Supplementary Information, Supplementary Fig. S4). As such, the possibility of artificially inflating the Mousterian component of the reconstituted EJOP sup sample is extremely unlikely. The overall analysed sample from EJOP sup comprises 4555 lithic artefacts greater than 1.5 cm, of which 694 were piece-plotted during excavations, a sample some 70% larger than previously reported (the complete database of piece-plotted material with original and reattributed level assignments is available as Dataset 1).

Additionally, Lévêque’s excavations notebooks (Supplementary Information, Supplementary Fig. S4) record the coordinates for what are, given the date, almost certainly the first 10 human remains, mainly teeth, exposed prior to the removal of the remaining skeletal elements in a 70-cm diameter plastered sediment block (Supplementary Information, Supplementary Fig. S5). These points therefore provide the uppermost limits of the Neanderthal skeletal material and allow us to reposition of this approximately 20 cm thick block of sediment in space.

### Techno-typological and taphonomical analysis of EJOP sup

In order to most reliably describe the techno-typological composition of EJOP sup and separate its components, lithic artefacts were designated as Châtelperronian or Middle Palaeolithic based on well-defined production objectives and morphological criteria available in the most recent and complete technological analyses of these techno-complexes. If objects did not fulfil either the well-established techno-typological criteria for the Châtelperronian^55–61^ or the various production methods or tool types typical of the Middle Palaeolithic^64–72^ they were left as ‘indeterminate’. For a table resuming the criteria for each diagnostic artefact class see Table S1 in the Supplementary Information. Additionally, we recorded macroscopic surface states and edge alterations for all lithic objects from the retained zone, complemented by a targeted analysis using 3D micrographs produced with a confocal microscope of a sample of material in the immediate vicinity of the Neanderthal skeletal remains (for the analytical protocol see Supplementary Information, Supplementary Figs 17–18).

## Results

**Figure 3:**
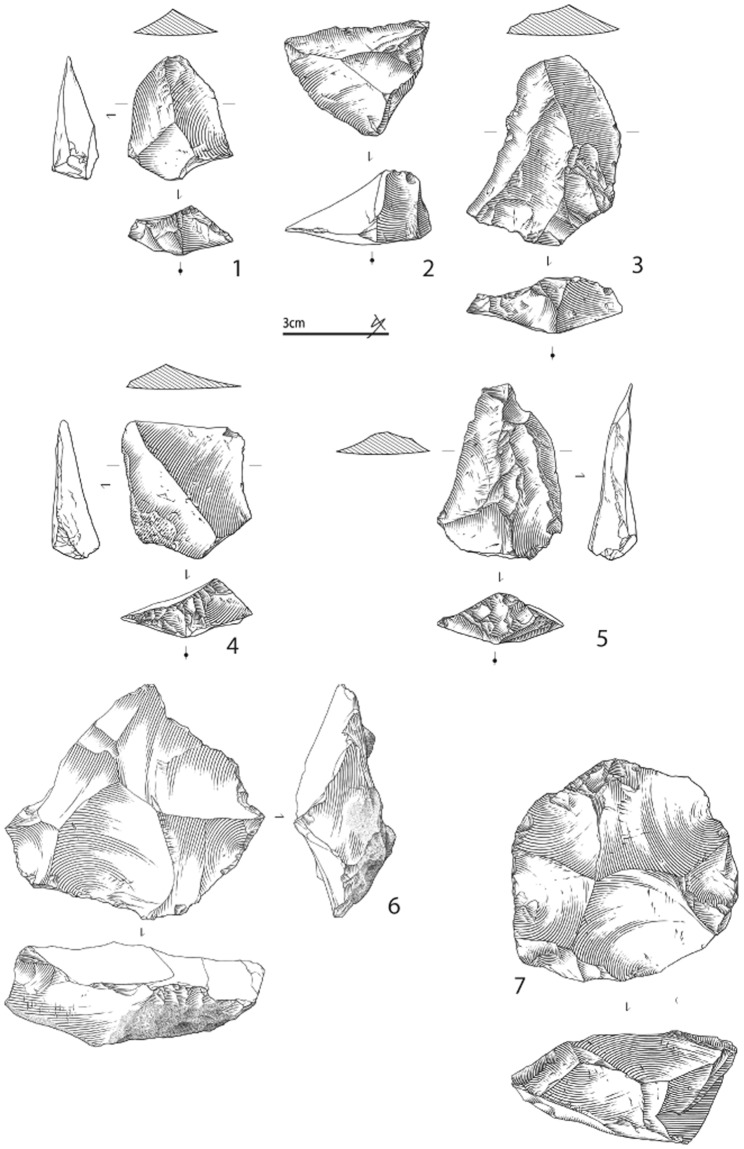
Pseudo-Levallois points (N° 1, 2, 4); éclat débordant (N° 3), Levallois flake (No. 5), Discoid cores (N° 6, 7) from the reattributed EJOP sup sample. Drawings S. Ducasse.

**Figure 4:**
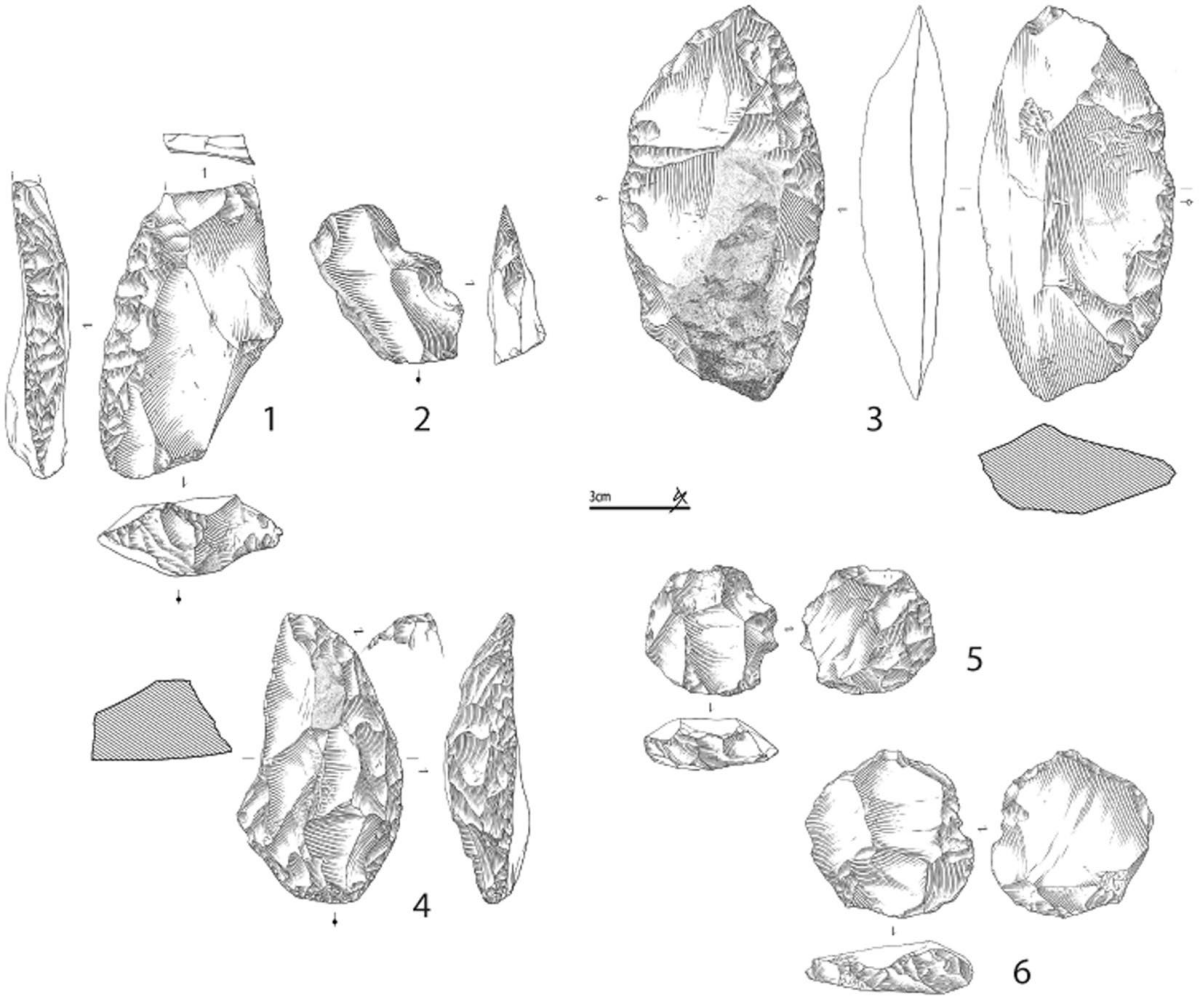
Large scraper with thinned back (N° 3), scraper on an éclat débordant (N° 1), denticulate (N° 2), half-Quina scraper (N° 4), Levallois cores (N° 5 and 6) from the reattributed EJOP sup sample. Drawings S. Ducasse.

Of the piece-plotted material attributed during excavations to EJOP sup (n = 696), 173 are diagnostic, and evince an assemblage composed primarily of Middle Palaeolithic cultural material (88.4% of the diagnostic pieces, n = 153) accompanied by a much smaller Châtelperronian component (11.6%, n = 20). The heavily Middle Palaeolithic character of this level is equally apparent with our re-attributed piece-plotted material (n = 975), with the proportions of diagnostic cultural material remaining relatively stable (Middle Palaeolithic, 84.6%, n = 237; Châtelperronian, 15.4%, n = 43). When the non-piece plotted material is included, these proportions shift only moderately, as more unretouched pieces are considered. Despite over 70% of the overall EJOP sup sample being broken or damaged, we were able to confidently assign 32% (n = 1471) to either the Middle Palaeolithic or Châtelperronian, evincing an assemblage comprising an extremely limited quantity of Châtelperronian cultural material (n = 88, 6%, including 12 Châtelperronian points) mixed with a considerably more substantial Middle Palaeolithic component (n = 1380, 94%, see Supplementary Information, Supplementary Tables S2 and S3). Clear evidence exists for use of the Levallois, Discoid and Kostenki/truncated-facetted flake production methods, accompanied by denticulates, notches, side-scrapers and a much larger number of pseudo-tools with mechanical retouch, a preservation pattern noted by Levêque himself (Figs 3 and 4). Photographs of the entire analysed lithic assemblage can be consulted in the Supplementary Information as Supplementary Figs 6–16. Finally, considering the strict selection criteria employed, it is important to note that the proportions of Middle Palaeolithic artefact types likely represent very conservative estimates. In the end, regardless the assemblage considered – either Levêque’s original attributions or our own, and whether only piece-plotted or all material is included – EJOP sup is best described as comprising two distinct techno-typological components: a dominant Middle Palaeolithic and an extremely limited Châtelperronian.

It is equally noteworthy that the Mousterian tool component of EJOP sup involves uniquely typical Mousterian blank types and not by-products of blade manufacture. As noted by Bachellerie^57^ and Roussel *et al*.^60^, respectively, for several open-air sites in the southwest of France and the Quinçay rockshelter sequence, Middle Palaeolithic type tools in Châtelperronian assemblages are made on by-products of blade production. At Saint-Césaire, Mousterian and Châtelperronian tools are produced on blanks following independent production methods, in other words, all Mousterian retouched tools are made on typical Mousterian flake blanks.

### Interpreting the mix of Mousterian and Châtelperronian components in EJOP sup

**Figure 5:**
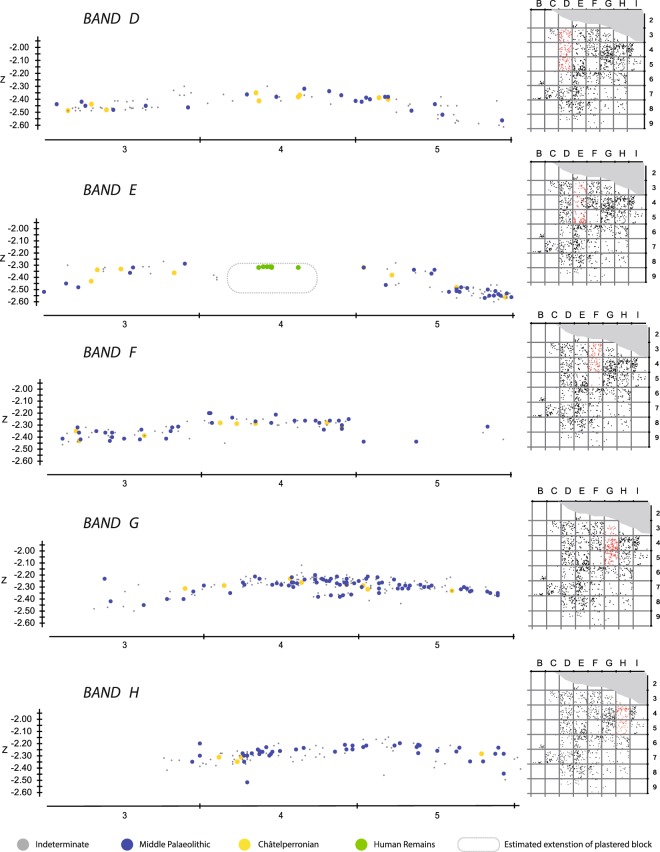
Projection of bands D through G of reattributed lithic material by chrono-cultural attribution including the position (band E) of the plastered block containing the Neanderthal remains that was removed and excavated in the laboratory.

We used spatial projections of piece-plotted material, surface alterations and the systematic conjoining of lithic fragments to explore the stratigraphic context of the two techno-typological components of EJOP sup. Detailed projections clearly reveal the layer’s Châtelperronian and Middle Palaeolithic components to be inseparable vertically or horizontally (Fig. 5) throughout the approximately 20 cm thick layer, with no clear lens of Châtelperronian material preserved at the summit. This pattern definitively rules out the possibility of a previously undetected stratigraphic superimposition of Mousterian and Châtelperronian lithic material within EJOP sup. As noted above, the clear presence of a sterile band between the underlying Mousterian level (EGPF and EJOP inf) and EJOP sup also effectively rules out the migration of Middle Palaeolithic material directly from the former into the latter (Fig. 2).

In terms of preservation, two thirds of the lithic material greater than 4 cm from EJOP sup portrays some form of edge damage (66%), with 14% bearing heavy alternate edge damaged, percussed ridges and lustred surfaces (Supplementary Information, Supplementary Table S4). This is in stark contrast with both the overlying Aurignacian and underlying EJOP inf and final Mousterian occupations^53,54,73^ and was clear to Lévêque^55^ himself. An earlier spatial analysis^74^ also demonstrated “naturally” modified pieces to cluster in the upslope area near the cliff, particularly in the central area (squares E4/5/6 and G4/5/6) where the Neanderthal skeleton was found. Importantly, and as previously suggested^53,54^, our analysis produced no clear correlation between surface states and the different techno-typological components of EJOP sup. In other words, the Mousterian and Châtelperronian material comprise both relatively fresh and heavily altered pieces. Furthermore, confocal microscopy reveals lithic artefacts in the vicinity of the Neanderthal skeletal remains to bear surface alterations directly comparable to those sampled from deposits in the immediate surroundings of the site (i.e. secondary slope deposits, colluvial deposits, see Supplementary Information, Supplementary Fig. S18). Roughness measurements show a wide variability of surface alterations among artifacts from an extremely limited volume (i.e., a 2 cm spit in a 50 by 50 centimeter sub-square), strengthening the idea that EJOP sup underwent multiple complex post-depositional processes that likely modified the spatial organization of the material.

**Figure 6:**
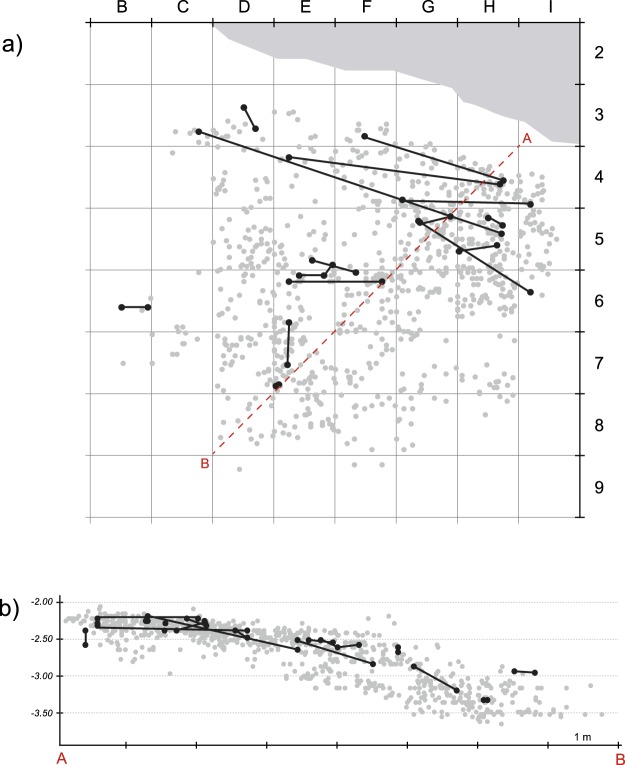
Vertical (B) and horizontal (A) projection of conjoined lithic artefacts (n = 29) from the systematic conjoining programme. The dotted line indicates the projection axis.

The systematic testing of conjoinable flake or blade fragments has been shown to be a highly efficient taphonomic tool for testing the displacement of both lithic and faunal material, and hence provides a clear means for assessing the potential post-depositional reworking of assemblages^74–76^. All broken pieces with complete breaks longer than 15 mm, including non-piece plotted artefacts, clearly identifiable as proximal, mesial and distal fragments from the entire excavated surface (32 sq. meters) and assigned to all the various stratigraphic distinctions of EJOP were systematically tested for break conjoins (See Supplementary Information for methodology, Supplementary Fig. S20). This entailed verifying nearly 700,000 possible connections between 1441 fragments (Supplementary Information, Supplementary Table S6, Fig. S19). In the end, only 29 connections (Supplementary Information, Supplementary Table S7), nearly all of which follow the double slope of the deposits (Fig. 6), were found for a success rate of 4%. Break conjoins involve an average artefact displacement ranging from several centimetres to several metres.

To our knowledge, this is the first time that a systematic break conjoining experiment has been applied to a context, archaeological or experimental, with a considerable flake component, meaning that comparable data is unfortunately lacking. However, several pertinent observations are still possible. First, the presence of a considerable number of cortical flakes and cortical fragments (primarily broken during detachment) in immediately available Senonian flint, alongside numerous cores and evidence for all stages of the various reduction sequences, plead in favour of a considerable portion of lithic production having been carried out on-site. Second, the excavated zone likely represents a large portion of the original occupation, making it extremely unlikely that all the missing fragments remain to be found in the limited area where EJOP sup is preserved in the upslope unexcavated zone. Third, the very limited number of intentional fractures observed during our technological analysis rules out a particular techno-economic behaviour whereby broken objects were consistently exported and/or imported.

Given this combination of arguments, it would be reasonable to anticipate a considerably higher success rate than the surprisingly low 4%. While the percentage of conjoins does not in and of itself demonstrate the disturbance of an archaeological layer, when compared to other systematic refitting analyses (see Supplementary Table S8), albeit from Upper Palaeolithic contexts, the success rate for Saint-Césaire is on the order of 2 to 3 times lower than demonstrably redistributed or mixed contexts (Corbiac-Vignoble II, Le Piage, Caminade) and nearly 9 times lower for the exceptionally well-preserved open-air site of Canaule II. Only the success rate for Roc de Combe is comparable; however, the excavated surface was considerably smaller than that of Saint Césaire.

Unlike previous refitting work on the faunal assemblages from Saint-Césaire, which focused on testing the inter-level movement of artefacts^77^, our systematic refitting programme was designed to address the preservation of connections within the various lithostratigraphic units of EJOP. While this previous analysis produced no evidence of any important post-depositional disturbance of the Saint-Césaire sequence, this uniquely concerned inter-level admixture and not the overall integrity and homogeneity of EJOP sup itself. This important difference in taphonomic approaches effectively accounts for what could be perceived as contradictory results between the two studies.

## Discussion and Conclusion

Our analysis shows EJOP sup to contain an extremely limited quantity of Châtelperronian cultural material clearly mixed with an overwhelmingly Middle Palaeolithic component. Moreover, these two assemblage components depict no relic stratigraphic superimposition whereby the Châtelperronian would occupy the summit of EJOP sup; both components (diagnostic non-retouched lithic material included) are fully intermixed. Arguments whereby EJOP sup would represent an ‘archaic Châtelperronian’ with a clear, substantially larger Mousterian component, as originally advanced by Lévêque^51^, would, based on recent analyses and the results presented here, not only require special pleading but be totally incongruent with well-contextualised, taphonomically-tested Châtelperronian occurrences that systematically lack a Mousterian component. In fact, recent comparisons of available tool counts from what were considered the most secure Châtelperronian contexts revealed EJOP sup to be the sole clear outlier in terms of its Mousterian tool component^36,57^. It is also instructive to note that the sidescrapers from EJOP sup (i.e. Fig. 4. n° 1, 3, 4.) find no equivalent in any known taphonomically-secure Châtelperronian context. In this same respect, the EJOP sup Mousterian component depicts absolutely no Upper Palaeolithic ‘tendencies’ (i.e., Upper Palaeolithic tool types, backed knives, elongated flake production, soft-hammer percussion) commonly advanced as support for a local Mousterian origin for the Châtelperronian^36,78^.

The most parsimonious explanation for the composition of the EJOP sup assemblage is the post-depositional redistribution of what was likely a very brief Châtelperronian stop-over, which have been shown to be frequent in the region^65,76^, within one or several different, considerably more consequential Mousterian occupations, themselves equally affected by these same processes. Several lines of evidence support this conclusion. First, the heavily reworked and mixed nature of this level is borne out by the condition of the assemblage itself. Second, the near total absence of break conjoins despite a systematic refitting program, which is equally compatible with results reported for the highly fragmented EJOP sup faunal component^77^, where of over 1000 tested specimens only 2 green bone fractures connections were found alongside four, less taphonomically-relevant dry bone fractures, all of which derive from the same sub-square. Finally, a diaclase, still evident in the cliff face (Supplementary Information, Supplementary Fig. S1), combined with the presence of several thousand geofacts of Santonian flint that outcrops directly above the site (Supplementary Information, Supplementary Fig. S21), strongly suggest the potential introduction of archaeological material from the overlying plateau. This likelihood is further supported by the confocal microscopy results and consistent with the double slope of the EJOP sup deposits, which would go some way in explaining the extremely small success rate for the systematic refitting programme.

Currently, apart from the mention of several intact articulations or elements in anatomical position, no spatial data is available concerning the precise organisation or bone taphonomy of the heavily fragmented and crushed Neanderthal skeletal material^44,45,79^. The reworking of the deposit was also likely responsible for the truncation, removal and or destruction of the partial Neanderthal skeleton, a probability reinforced by the fact that the majority of the naturally modified lithic material was found in the area containing the skeleton^80^. It is equally interesting to note that, while practically the entire left cranial-mandibular block is missing^44,45^, several teeth reported in the excavation notebooks (see Supplementary Information, Supplementary Information Fig. S5) as left molars and premolars were found clustered near the majority of the skeletal material. This would suggest that at least the other half of the skull, if not the missing infra-cranial elements, were probably originally present, rendering the hypothesis of a secondary burial of a partial skeleton^45^ difficult to maintain. With this being the case, three potential scenarios for the deposition and hence chrono-cultural association of the Neanderthal skeletal remains can be imagined; either they were deposited (1) subsequent to the reworking event and are thus Aurignacian in age^81^, (2) they were associated with a brief Châtelperronian occupation, or (3) they represent the remnants of a reworked Mousterian ‘burial’.

In the post-reworking (Aurignacian) scenario, we would expect the sediments removed with the plastered block, and perhaps, the immediately surrounding sediment to potentially contain some Aurignacian cultural material. This is not the case - the block contained an extremely limited number of lithics, with all diagnostic material being techno-typologically Mousterian in nature (Supplementary Table S5). Moreover, the lack of stones in the ‘burial’ area supporting the presence of the pit^81^ is irrelevant in that multiple, similar-sized areas lacking stones were documented across the excavated surface of EJOP sup^80^. Finally, this scenario, although not impossible, would be inconsistent with almost all current models for the Middle-to-Upper Palaeolithic transition in Western Europe, where anatomically modern human groups are commonly assumed to be the authors of the Aurignacian (see^82^ for a recent synthesis).

**Figure 7:**
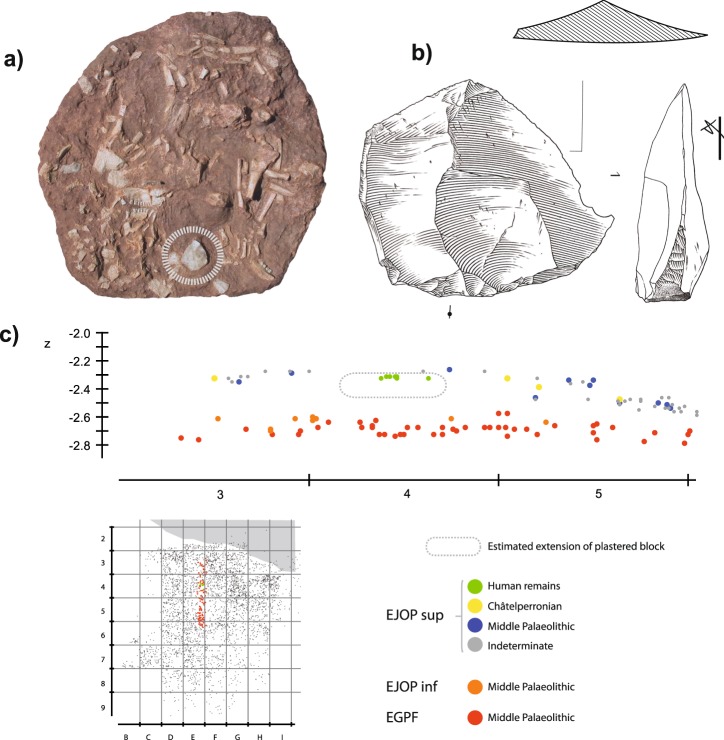
Photo of the moulded block of Neanderthal skeletal remains (upper right) and large éclat débordant found immediately to the right of the mandible (upper left). Drawings S. Ducasse. Projection (middle) of an approximately 40 cm band through area containing the human remains (bottom right). Note that the first human remains discovered lie slightly below the piece-plotted lithic material.

Separating the second two scenarios (a Châtelperronian versus Mousterian association) based on the evidence available would also appear highly speculative. The summit of the sediment block containing the Neanderthal remains does, however, appears to lie towards the base of EJOP sup or at the summit of the sterile band (Fig. 7), which would be compatible with the extremely limited quantity of lithic material recovered from the plastered block. This potentially suggests the introduction of the remains to pre-date the reworking event (or events) that would have partially disturbed the skeleton, eroded the summit of EJOP sup and mixed the overlying archaeological material. With this being the case, associating the remains with either cultural component is impossible. With that said and to be as clear as possible, our argument is not that because the Neanderthal skeleton cannot be reliably associated with Châtelperronian cultural material; ergo, it must be Mousterian, rather we maintain that faced with a clearly reworked archaeological context and significant ambiguity surrounding when the Neanderthal skeletal material was introduced, it is impossible to advance any reliable chrono-cultural association whatsoever.

In sum, given the demonstrably mixed nature of EJOP sup and the impossibility of attributing the Neanderthal remains to a given techno-complex, the Neanderthal-Châtelperronian association at Saint Césaire should be considerable unreliable at best. Any Châtelperronian affiliation would therefore be based uniquely on a sole radiocarbon age obtained from the Neanderthal skeleton^33^. However, given both the low collagen content of the sample coupled with persistent challenges obtaining reliable dates from this period^83,84^, this age should be viewed with considerable caution. New excavations, which include a concerted geo-archaeological and micro-morphological approach, should help shed critical new light on the complex sedimentary and post-depositional processes responsible for the formation of EJOP sup. The example of Saint Césaire ones again highlights how a detailed taphonomic analysis can significantly impact both interpretations and their relevance for sociocultural models for the Middle-to-Upper Palaeolithic transition in Western Europe.

## Electronic supplementary material

Supplementary Information

Supplementary Dataset 1

## References

[1] Henshilwood C, d’Errico F, Vanhaeren M, van Niekerk K, Jacobs Z (2004). Middle Stone Age Shell Beads from South Africa. Science.

[2] Henshilwood C, d’Errico F, Watts I (2009). Engraved ochres from the Middle Stone Age levels at Blombos Cave, South Africa. J Hum Evol..

[3] Henshilwood C (2009). A 100,000-Year-Old Ochre-Processing Workshop at Blombos Cave, South Africa. Science.

[4] d’Errico F, Henshilwood C, Vanhaeren M, van Niekerk K (2005). Nassarius kraussianus shell beads from Blombos Cave: evidence for symbolic behaviour in the Middle Stone Age. J. Hum Evol..

[5] d’Errico F (2009). Additional evidence on the use of personal ornaments in the Middle Paleolithic of North Africa. Proc. Natl Acad Sci..

[6] d’Errico F (2012). Early evidence of San material culture represented by organic artifacts from Border Cave, South Africa. Proc. Natl Acad Sci..

[7] Bouzouggar A (2007). 82,000-year-old shell beads from North Africa and implications for the origins of modern human behavior. Proc. Natl Acad Sci..

[8] Brown KS (2009). Fire As an Engineering Tool of Early Modern Humans. Science.

[9] Wadley L, Hodgskiss T, Grant M (2009). Implications for complex cognition from the hafting of tools with compound adhesives in the Middle StoneAge, South Africa. . Proc. Natl Acad Sci..

[10] Mourre V, Villa P, Henshilwood C (2010). Early Use of Pressure Flaking on Lithic Artifacts at Blombos Cave, South Africa. Science.

[11] Texier P-J (2010). A Howiesons Poort tradition of engraving ostrich eggshell containers dated to 60,000 years ago at Diepkloof Rock Shelter, South Africa. Proc. Natl Acad Sci..

[12] Texier P-J (2013). The context, form and significance of the MSA engraved ostrich eggshell collection from Diepkloof Rock Shelter, Western Cape, South Africa. J. Archaeol Sci..

[13] Soressi M (2013). Neandertals made the first specialized bone tools inEurope. Proc. Natl Acad Sci..

[14] Costamagno, S. & Tartar, E. L’utilisation des matières osseuses au Moustérien in *Neandertal à la loupe* (eds Turq, A., Faivre, J.-Ph., Maureille, B., Lahaye, Ch. & Bayle, P.) 89–96 (RMN, 2016).

[15] Soressi, M. & d’Errico, F. Pigments, gravures, parures: Les comportements symboliques controversés des Néandertaliens *Les* Néandertaliens Biologie *et cultures* (eds Vandeermersch, B. & Maureille, B.) 297–309 (CTHS Editions, 2007).

[16] Soressi, M. *et al*. Pech-de-l’Azé I (Dordogne, France): nouveau regard sur un gisement moustérien de tradition acheuléenne in *Les sociétés Paléolithiques d’un grand Sud-Ouest: nouveaux gisements, nouvelles méthodes, nouveaux résultats* (eds Jaubert, J., Bordes, J. G. & Ortega, I.) 95–132 (Société Préhistorique française, 2008).

[17] Roebroeks W (2012). Use of red ochre by early Neandertals. Proc. Natl Acad Sci.

[18] Bonjean D (2015). A new Cambrian black pigment used during the late Middle Palaeolithic discovered at Scladina Cave (Andenne, Belgium). J. Archaeol Sci..

[19] Arensburg B (1985). A Neanderthal burial in the Kebara Cave, Israel. Compte Rendu l’Académie des Sci Paris..

[20] Petitt, B. *The Palaeolithic Origins of Human Burial* (Routledge, 2011).

[21] Rendu W (2014). Evidence supporting an intentional Neandertal burial at La Chapelle-aux-Saints. Proc. Natl Acad Sci..

[22] Rendu W (2016). Let the dead speak…comments on Dibble *et al*.’s reply to “Evidence supporting an intentional burial at La Chapelle-aux-Saints”. J. Archaeol Sci..

[23] Gargett R (1989). Grave Shortcomings: The Evidence for Neandertal Burial. Curr. Anthropol..

[24] Sandgathe DM, Dibble HL, Goldberg P, McPherron SP (2011). The Roc de Marsal Neandertal child: a reassessment of its status as a deliberate burial. J. Hum Evol..

[25] Dibble HL (2015). A critical look at evidence from La Chapelle-aux-Saints supporting an intentional Neandertal burial. J Archaeol Sci..

[26] Zilhão J (2010). Symbolic use of marine shells and mineral pigments by Iberian Neandertals. Proc. Natl Acad Sci..

[27] Morin E, Laroulandie V (2012). Presumed symbolic use of diurnal raptors by Neanderthals. PLoS One..

[28] Peresani M, Vanhaeren M, Quaggiotto E, Queffelec A, d’Errico F (2013). An Ochered Fossil Marine Shell From the Mousterian of Fumane Cave, Italy. PLoS One..

[29] Romandini M (2014). Convergent evidence of eagle talons used by late Neanderthals in Europe: A further assessment on symbolism. PLoS One..

[30] Klein, R. *Ice-Age Hunters of the Ukraine* (University of Illinois Press, 1973).

[31] Otte, M. From the middle to the upper Palaeolithic: the nature of the transition in *The Emergence of Modern Humans: An Archaeological Perspective* (ed. Mellars, P) 483–456 (Cambridge University Press, 1990).

[32] Hublin JJ, Spoor F, Braun M, Zonneveld F, Condemi S (1996). A late Neanderthal associated with Upper Palaeolithic artefacts. Nature.

[33] Hublin JJ (2012). Radiocarbon dates from the Grotte du Renne and Saint-Cesaire support a Neandertal origin for the Chatelperronian. Proc. Natl Acad Sci.

[34] Mellars, P. *The Neanderthal Legacy: An Archaeological Perspective from Western* (Princeton University Press, 2006).

[35] Mellars P (2005). The impossible coincidence. A single-species model for the origins of modern human behavior in Europe. Evol. Anthropol..

[36] Ruebens K, McPherron SJP, Hublin JJ (2015). On the local Mousterian origin of the Châtelperronian: Integrating typo-technological, chronostratigraphic and contextual data. J. Hum Evol..

[37] d’Errico F, Zilhão J, Julien M, Baffier D, Pelegrin J (1998). Neanderthal Acculturation in Western Europe*?*. Curr. Anthropol..

[38] d’Errico F (2005). The invisible frontier. A multiple species model for the origin of behavioral modernity. Evol Anthropol..

[39] Zilhão J, Errico F (1999). The Chronology and Taphonomy of the Earliest Aurignacian and Its Implications for the Understanding of Neandertal Extinction. J. of World Prehist..

[40] Zilhão J (2006). Neandertals and Moderns Mixed, and It Matters. Evol. Anthropol..

[41] Leroi-Gourhan A (1958). Etude des vestiges humains fossiles provenant des grottes d’Arcy-sur-Cure. Annales de Paléontologie..

[42] Welker F (2016). Palaeoproteomic evidence identifies archaic hominins associated with the Châtelperronian at the Grotte du Renne. Proc. Natl Acad Sci..

[43] Lévêque F, Vandermeersch B (1980). Les découvertes de restes humains dans un horizon castelperronien de Saint-Césaire (Charente-Maritime). Bull. de la Soc. Préhist. de Française..

[44] Vandermeersch B (1984). A propos de la découverte du squelette néandertalien. Bulletins et Mémoires de la Société d’anthropologie de Paris.

[45] Vandeermersch, B. Was the Saint-Césaire Discovery a Burial in *Context of a late Neandertal: Implications of multidisciplinary research for the transition to Upper Paleolithic adaptations at Saint Césaire, Charente-Maritime, France* (eds Lévêque, F., Backer, A. M. & Guibaud, M.) 129–131 (Préhistory Press, Madison, 1993).

[46] Bordes F (1981). Neandertal Encombrant. La Recherche.

[47] Sonneville-Bordes D (1989). Préface. Bulletin de la Société Préhistorique de l’Ariège.

[48] Bar-Yosef O (2007). The archaeological framework of the Upper Paleolithic revolution. Diogenes..

[49] Bar-Yosef O, Bordes J-G (2010). Who were the makers of the Châtelperronian culture?. J Hum Evol.

[50] Bordes J-G, Teyssandier N (2011). The Upper Paleolithic nature of the Châtelperronian in South-Western France: Archeostratigraphic and lithic evidence. Quat. Intl..

[51] Lévêque, F. The Castelperronian Industry of Saint-Césaire: The Upper Level. In *Context of a late Neandertal: Implications of multidisciplinary research for the transition to Upper Paleolithic adaptations at Saint Césaire, Charente-Maritime, France* (eds Lévêque, F., Backer, A. M. & Guibaud, M.) 25–35 (Préhistory Press, Madison, 1993).

[52] Guilbaud, M. Debitage from the Upper Castelperronian Level at Saint-Césaire. In *Context of a late Neandertal: Implications of multidisciplinary research for the transition to Upper Paleolithic adaptations at Saint Césaire, Charente-Maritime, France* (ed. Lévêque, F., Backer, A. M. & Guibaud, M.) 39–58 (Préhistory Press, Madison, 1993).

[53] Soressi, M. La Roche-à-Pierrot à Saint-Césaire (Charente-Maritime). Nouvelles données sur l’industrie lithique du Châtelperronien In *Préhistoire entre Vienne et Charente. Hommes et sociétés du Paléolithique. 25 ans d’archéologie préhistorique en Poitou-Charentes* (eds Buisson-Cattil, J. & Primault, J.) 191–201 (Association des Publications Chauvinoises, 2011).

[54] Soressi M (2011). Révision taphonomique et techno-typologique des deux ensembles attribués au Châtelperronien de la Roche-à-Pierrot à Saint-Césaire. L’Anthropologie.

[55] Pelegrin, J. Technologie lithique: le Châtelperronien de Roc-de-Combe (Lot) et de La Côte (Dordogne). (Edition du CNRS, 1995).

[56] Connet, N. Le Châtelperronien, réflexions sur l’unité et l’identité techno-économique de l’industrie lithique:l’apport de l’analyse diachronique des industries lithiques des couches châtelperroniennes de la grotte du Renne à Arcy-sur-Cure (Yonne). (Unpublished Doctoral Dissertation, Lille University, 2002).

[57] Bachellerie, F. Quelle unité pour le Châtelperronien? Apport de l’analyze taphonomique et techno-économique des industries lithique de trois gisements aquitains de plein air: le Basté, Bidart (Pyrénées-Atlantiques) et Canaule II (Dordogne). (Unpublished Doctoral Dissertation. University of Bordeaux, 2011).

[58] Roussel M (2011). Méthodes et rythmes du débitage laminaire au Châtelperronien: comparaison avec le Protoaurignacien. C. R Palevo.l.

[59] Baillet M, Bachellerie F, Bordes JG (2014). Enquête autour d’un outil: approche techno-économique, fonctionnelle et expérimentale des grattoirs châtelperroniens de Canaule II (Creysse, Dordogne, France). Paléo.

[60] Roussel M, Soressi M, Hublin J-J (2015). The Châtelperronian conundrum: Blade and bladelet lithic technologies from Quinçay, France. J. Hum Evol..

[61] Bodu P (2017). Un gisement châtelperronien de plein air dans le Bassin parisien: Les Bossats à Ormesson(Seine-et-Marne). Gallia Préhistoire.

[62] Rigaud, J. P. Late Neandertals in the South West of France and the emergence of the Upper Paleolithic in *Neanderthals on the Edge: 150th Anniversary Conference of the Forbes’ Quarry Discovery, Gibraltar* (eds Stringer, C. B., Barton, N. & Finlayson, C.) 27–31 (Oxbow Books, 2000).

[63] Bachellerie F, Bordes JG, Morala A, Pelegrin J (2007). Étude typo-technologique et spatiale de remontages lithiques de Canaule II, site châtelperronien de plein-air en Bergeracois (creysse, Dordogne). Paléo..

[64] Boëda E (1993). Le débitage discoïde et le débitage Levallois récurrent centripète. Bulletin de la Société Préhistorique Française..

[65] Baillet, M. *Eclairage de la tracéologie lithique sur le système nomade châtelperronien*. (Unpublished Doctoral Dissertation, University of Bordeaux, 2017).

[66] Boëda, E. *Le concept Levallois: variabilité des méthodes*. (CNRS Editions, 1994).

[67] Boëda E, Geneste JM, Meignen L (1990). Identification de chaînes opératoires lithiques du Paléolithique ancien et moyen. Paléo..

[68] Delagnes, A. L’organisation de la production lithique au Paléolithique moyen: approche technologique à partir des industries de La Chaise-de-Vouthon (Charente). (Doctoral Dissertation, Université de Paris X Nanterre, 1992).

[69] Locht JL, Swinnen C (1994). Le débitage discoïde du gisement de Beauvais (Oise): aspects de la chaîne opératoire au travers de quelques remontages. Paléo..

[70] Jaubert J, Mourre V (1994). Coudoulous, Le Rescoundudou, Mauran: diversité des matières premières et variabilité des schémas de production d’éclats. Quat. Nov..

[71] Peresani, M. *Discoid Lithic Technology: Advances and Implications*. (Archaeopress, 2003).

[72] Faivre JP, Gravina B, Bourguignon L, Discamps E, Turq A (2017). Late Middle Palaeolithic lithic technocomplexes (MIS 5–3) in the northeastern Aquitaine Basin: Advances and challenges. Quat Int..

[73] Thiébaut C, Meignen L, Levêque F (2009). Les dernières occupations moustériennes de Saint-Césaire (Charente-Maritime, France). Bulletin de la Société Préhistorique Française..

[74] Tixier, J. *Méthode pour l’étude des outillages lithiques*. (Unpublished Doctoral Dissertation. Université de Paris X, 1978).

[75] Bordes, J.-G. Les interstratifications Châtelperronien/Aurignacien du Roc-de-Combe et du Piage (Lot, France). Analyse taphonomique des industries lithiques; implications archéologiques (Unpublished Doctoral Dissertation, University of Bordeaux, 2002).

[76] Bachellerie F (2011). Archaeological Signatures of Hunting Activities Applied to Comparisonsn of Mousterian, Châtelperronian and Aurignacian Industries in the Pyrenees: The Nature of Hunting Tools and Site Functions. Palethnologie..

[77] Morin E (2005). Bone refits in stratified deposits: testing the chronological grain at Saint-Césaire. J. Archaeol Sci..

[78] Pelegrin, J. & Soressi, M. Le Châteperronien et ses rapports avec le mousterien. Une aire géographique relativement restreinte, une position chronologique récemment précisée in *Les Néanderthaliens: Biologie et Culture* (eds Vandeermeersch, B. & Maureille, B.) 283–296 (CTHS, 2007).

[79] Vandermeersch, B. & Hublin, J. J. Les Derniers Neanderthaliens in *Les Néanderthaliens: Biologie et Culture* (eds Vandeermeersch, B. & Maureille, B.) 109–115 (CTHS, 2007).

[80] Backer, A. M. Spatial Distributions at La Roche à Pierrot, Saint-Césaire: Changing Uses of a Rockshelter in *Context of a late Neandertal: Implications of multidisciplinary research for the transition to Upper Paleolithic adaptations at Saint Césaire, Charente-Maritime, France* (eds Lévêque, F., Backer, A. M. & Guibaud, M.) 105–127 (Préhistory Press, Madison, 1993).

[81] Zilhão, J. Neandertal-Modern Human Contact in Western Eurasia: Issues of Dating, Taxonomy, and *Cultural Associations in Dynamics of Learning in Neanderthals and Modern Humans Volume 1: Cultural Perspectives* (eds Akazawa, T., Nishiaki, K. & Aoko. Y.) 21–57 (Springer, 2013).

[82] Hublin JJ (2015). The modern human colonization of western Eurasia: when and where?. Quat. Sci Rev..

[83] Devièse T (2017). Direct dating of Neanderthal remains from the site of Vindija Cave and implications for the Middle to Upper Paleolithic transition. Proc. Natl Acad Sci. USA.

[84] Hublin JJ (2017). The last Neanderthal. Proc. Natl Acad Sci. USA.

