# Supplementary material for: No Reliable Evidence for a Neanderthal-Châtelperronian Association at La Roche-à-Pierrot, Saint-Césaire
Source: Sci Rep. 2018 Oct 11;8:15134. doi: 10.1038/s41598-018-33084-9 (PMC6181958; doi:10.1038/s41598-018-33084-9)
Supplement: Supplementary file 1 — Supplementary Information [file 41598_2018_33084_MOESM1_ESM.pdf]

No Reliable Evidence for a Neanderthal-Châtelperronian Association at La-Roche-à-Pierrot, Saint-Césaire

Brad Gravina, François Bachellerie, Solène Caux, Emmanuel Discamps, Jean-Philippe Faivre, Aline Galland, Alexandre Michel, Nicolas Teyssandier and Jean-Guillaume Bordes

SUPPORTING INFORMATION

Site location and geomorphological context (fig. S1) 2

Excavation history (fig. S2) 3

Stratigraphy and chrono-cultural attributions 5

Stratigraphic Reattributions (fig. S3) 6

Excavation notebook (fig. S4-5) 8

Techno-typology of diagnostic lithic artefacts (tabl. S1) 9

Tool counts, surface alterations, and artefact photos (tabl. S2-5, fig. S6-16) 10

Analytical protocol for the evaluation of surface alterations using confocal microscopy (A. Galland, fig. S17-18) 19

Systematic testing for conjoinable fragments (tabl. S6-S7, fig. S19-20) 22

Comparative data for systematic testing of break conjoins (tabl. S8) 25

Lithic raw materials (S. Caux, fig. S21) 26

Bibliography 28

Site location and geomorphological context (fig. S1)

The site of Roche à Pierrot is located on the commune of Saint Césaire, a dozen kilometres east of Saintes in the Charente-Maritime department of south-western France (Fig. S1, upper left). This now collapsed rock shelter lies at the base of a 5 to 6 m high Upper Turonian limestone cliff on the right bank of the Coran, a small tributary of the Charente River (Fig. S1, upper left). A fissure (diaclase) is still visible in the cliff face (see digital elevation model in Fig S1, bottom).

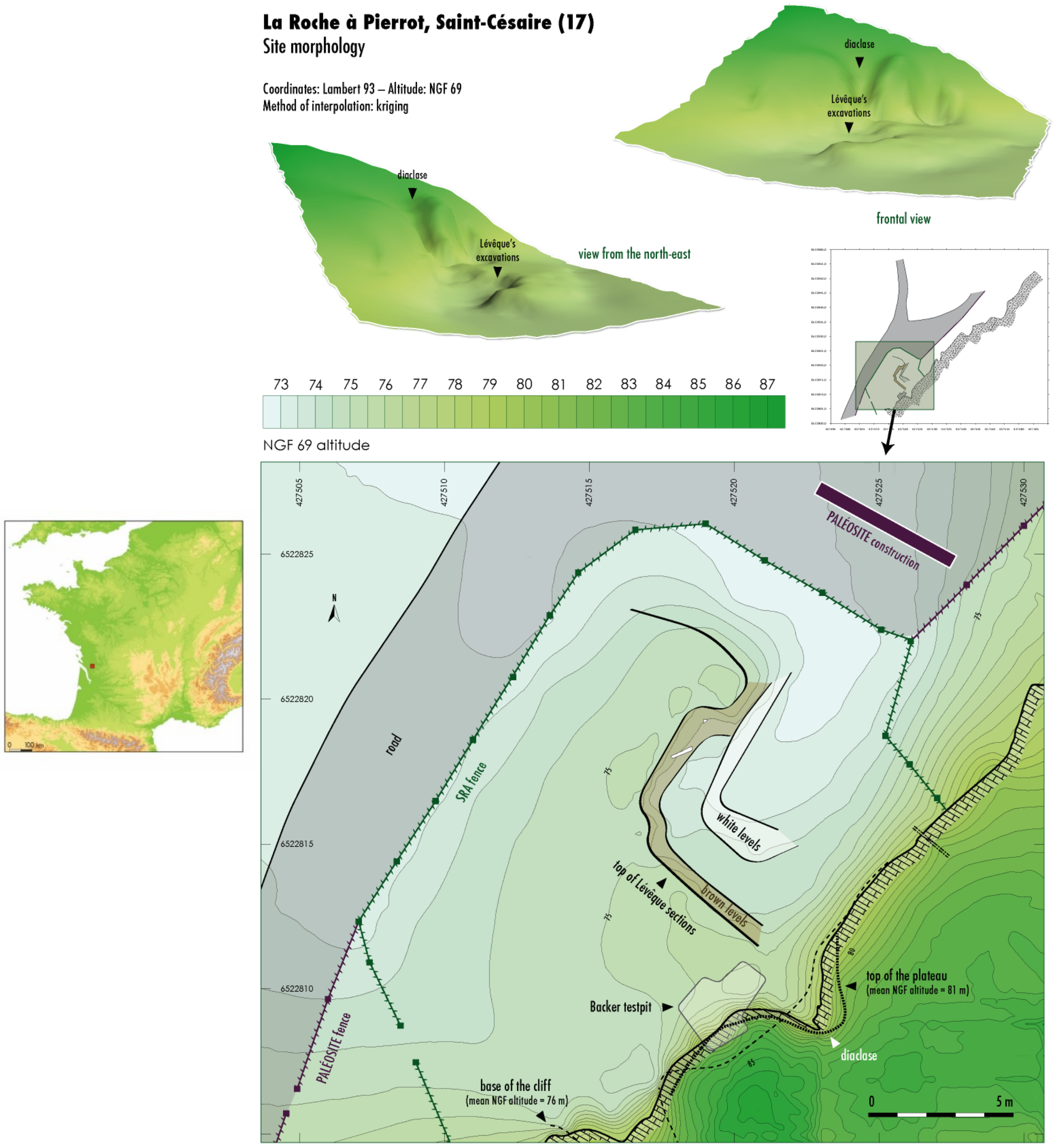

FIGURE S1: La Roche à Pierrot. Location (middle left) and digital elevation model of the site following Lévêque's excavations, note the fissure in the cliff face. (Illustration in Adobe Illustrator by F. Lacrampe-Cuyaubère – Archéosphère)

Excavation history (fig. S2)

The white Turonian limestone had been quarried until the end of the 20th century, producing numerous subterranean galleries within the surrounding cliffs and atop the plateau overlooking the site. The majority of these galleries were subsequently reused for the cultivation of mushrooms. The site of Roche-à-Pierrot was discovered during terracing work designed to create access to one of these mushroom farms. Although partially destroyed during this work, Francois Lévêque directed an 11 years excavation over a total of 52 m² that ended in 1987 (Fig. S2). A.M. Backer subsequently directed a small excavation in 1993 with the intermittent help of Lévêque. This work focused essentially on squares D8 and E8 in order to verifier the continuity of EJOP sup in this area of the site. Details concerning excavation methods employed at Saint-Césaire can be found in Lévêque (2002) and here we summarize only the most pertinent aspects.

The site was excavated using a one square meter grid system divided into 4 sub-squares. Objects were not systematically piece-plotted; only those considered to « present some archaeological, stratigraphic or paleontological interest » (Lévêque 2002: 418). The coordinates of piece-plotted objects were recorded in excavation notebooks for each square and subsequently marked on the pieces themselves following very light washing (see Fig. S4 for an example). Non piece-plotted material was for the most part recorded by square and sub-square and generally collected by 5 or 10 cm spits (for example, spit 34 would comprise sediments from a depth of between 331 and 340 cm below datum and indicated on the labels as 34 with an upper and lower bar). This is for example the case with the area directly surrounding the plastered cast containing the Neanderthals remains. The excavation notebooks equally indicate non-piece plotted material, which corresponds to no particular size class cut-off, to have been recorded uniquely by 20 cm spits *in the area surrounding the skeletal material* (see Fig. S5).

At the time of analysis, apart from several objects (including the typical cordiform biface) in the display cases of the Musée des antiquités nationales (Paris) and the piece-plotted objects, the near totality of non-piece plotted material was found unwashed and unmarked in the stores of the DRAC Poitou-Charente in Poitiers. The lithic material was subsequently washed, marked, counted and entered into a database alongside the available stratigraphic information provided on the accompanying labels.

A second rescue excavation directed by A.M. Backer in 1997 addressed only lines 8 and 9 and aimed to (1) assess the condition of the site, (2) clean the collapsed and walked-upon witness sections, (3) produce section drawings of the intact archaeological levels, (4) temporarily stabilise and protect the site, and (5) judge the best manner to protect the site more permanently.

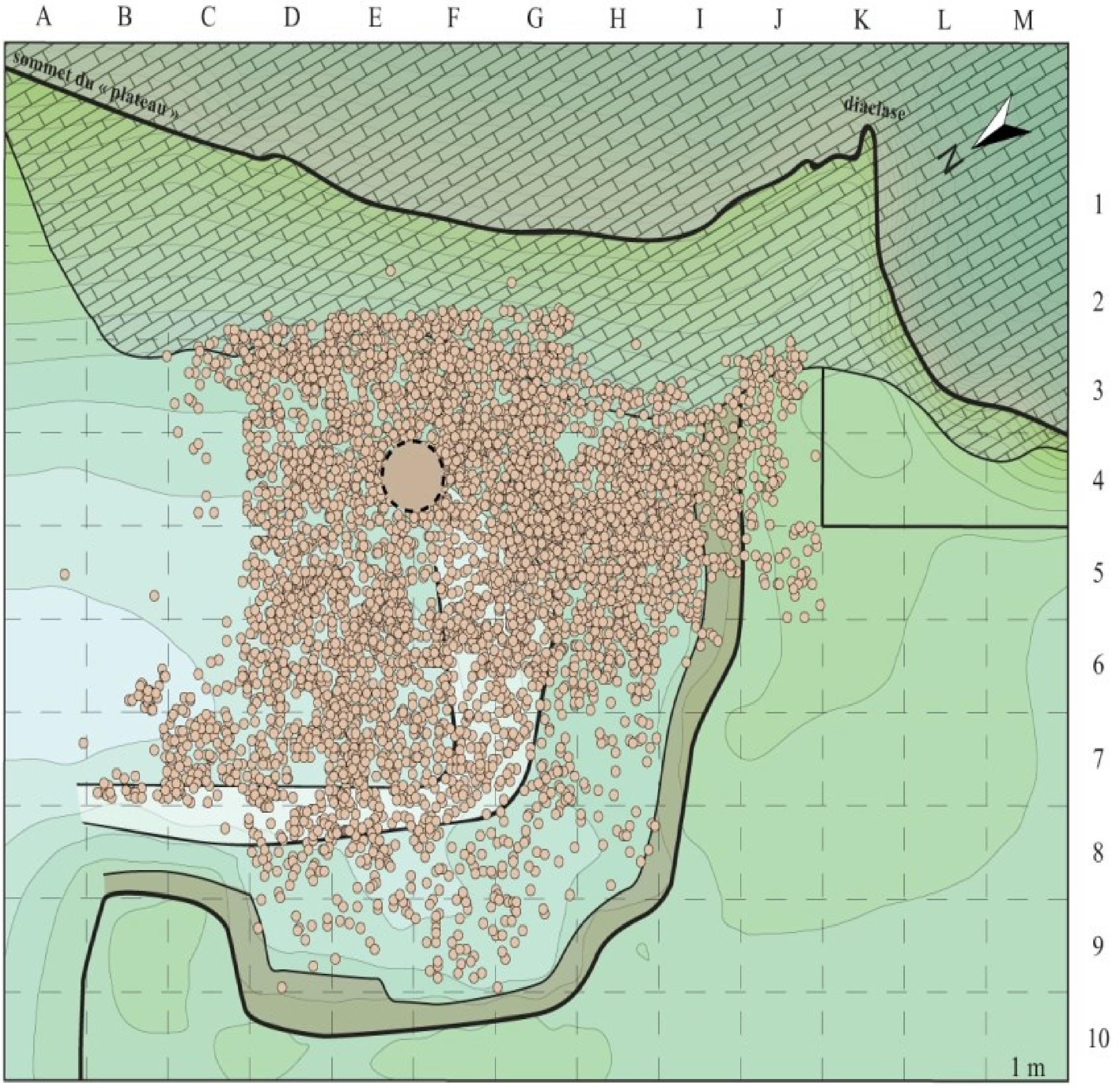

FIGURE S2: Roche à Pierrot, totality of piece plotted artefacts from all EJOP stratigraphic designations during Lévêque's excavations. The circle indicates the position of the Neanderthal skeleton in EJOP sup.

Stratigraphy and chrono-cultural attributions

The stratigraphy of Roche-à-Pierrot is separated into three principal sedimentary units (details can be found in Lévêque, Backer and Guilbaud, 1993) (Fig. S3):

- The **ensemble jaune** (EJ) sequence comprises six levels attributed to the Upper Palaeolithic
  - EJJ: Evolved Aurignacian
  - EJM: Evolved Aurignacian
  - EJF: Early Aurignacian
  - EJO sup: Protoaurignacian
  - EJOP sup: Châtelperronian
  - EJOP inf: initially assigned to the Châtelperronian, now to the Mousterian (Soressi, 2010).
- The **ensemble gris** (EG): five Middle Palaeolithic levels
  - EGPF: Discoid-Denticulate Mousterian (Thiébaut et al., 2009)
  - EGP: Denticulate Mousterian
  - EGF: Denticulate Mousterian
  - EGC: Mousterian of Acheulean Tradition (MTA)
  - EGB sup: Mousterian of Acheulean Tradition (MTA)
- The **ensemble rouge** (ER): sterile red clays level resting on the bedrock.

Stratigraphic Reattributions (fig. S3)

The manner in which the undifferentiated EJOP material was reattributed (when possible) to either EJOP sup or inf and was carried out as follows:

(1) A database of piece-plotted material of all material from all the various EJOP stratigraphic designations (EJOP, EJOP sup, EJOP inf, etc) was created using coordinates, square, sub-square and spit altitude marked on the pieces during excavations (Fig. S4, bottom right). The coordinates of all Châtelperronian points were crosschecked against those recorded in the excavations notebooks.

(2) This material was subsequently projected using the DataDesk© software suite by 20 cm slices in sagittal and frontal view by sub-square (Fig. S4).

(3) We then compared the vertical distribution of the projected material by level attribution (Fig. S4), reassigning pieces identified as EJOP to either EJOP inf or EJOP sup only when they fell clearly *within* the vertical distribution of pieces-plotted material assigned to either of these two sub-levels *during excavations*. Artefacts with uncertain stratigraphic positions (i.e. not squarely falling within the limits of either EJOP sup or inf were left as EJOP and excluded from analysis).

(4) The newly defined vertical limits of these sub-levels in each sub-square equally formed the basis for reassigning non-piece plotted material. As explained in the main text and readily apparent from the example provided below (figure S4), the fact that the lower limits of the lowermost spit assigned to EJOP sup fall in the sterile band separating EJOP sup from EJOP inf effectively eliminates any possibility of artificially inflating the Mousterian component of the analysed sample.

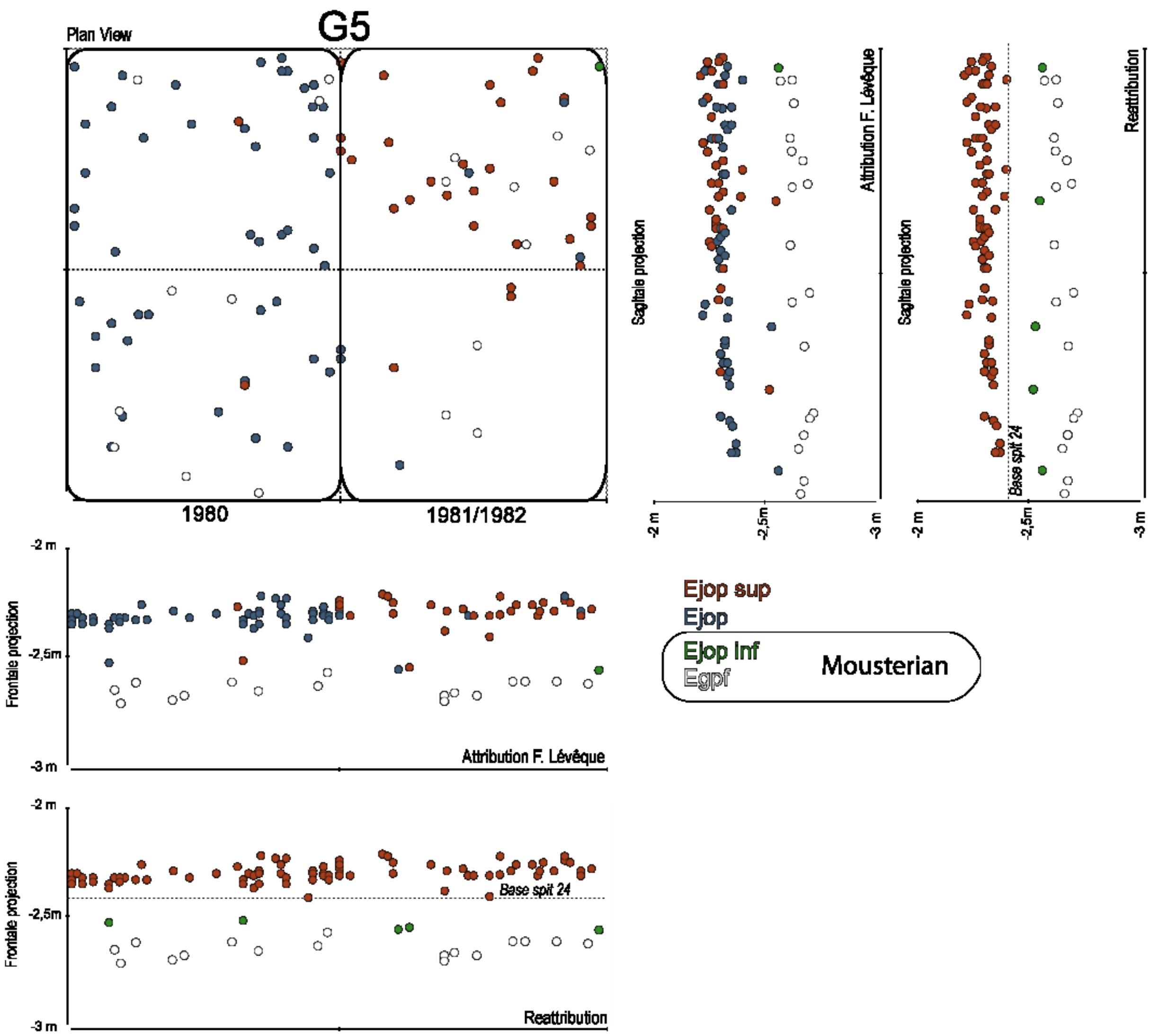

FIGURE S3: Example of compared vertical distribution by 50 cm x 50 cm sub-squares and piece-plotted artefact with coordinates, spit, square, sub-square and level. Note that EJOP sup was only identified during excavations from 1981/1982 onwards and that the lower limits of the lowermost spit assigned to EJOP sup fall within the sterile band

Excavation notebook (fig. S4-5)

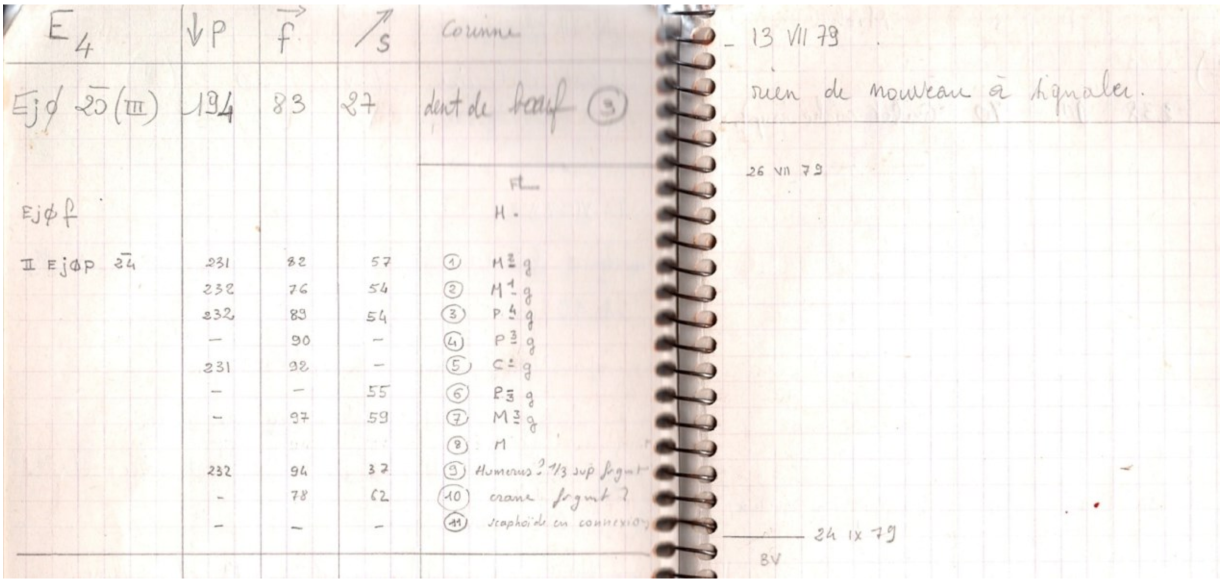

FIGURE S4: Scan of a page from Lévêque's field notebooks for square E4 with the coordinates for the first human remains recovered, primarily left dental elements (e.g. M2g, P3g,), prior to the removal of the plastered block.

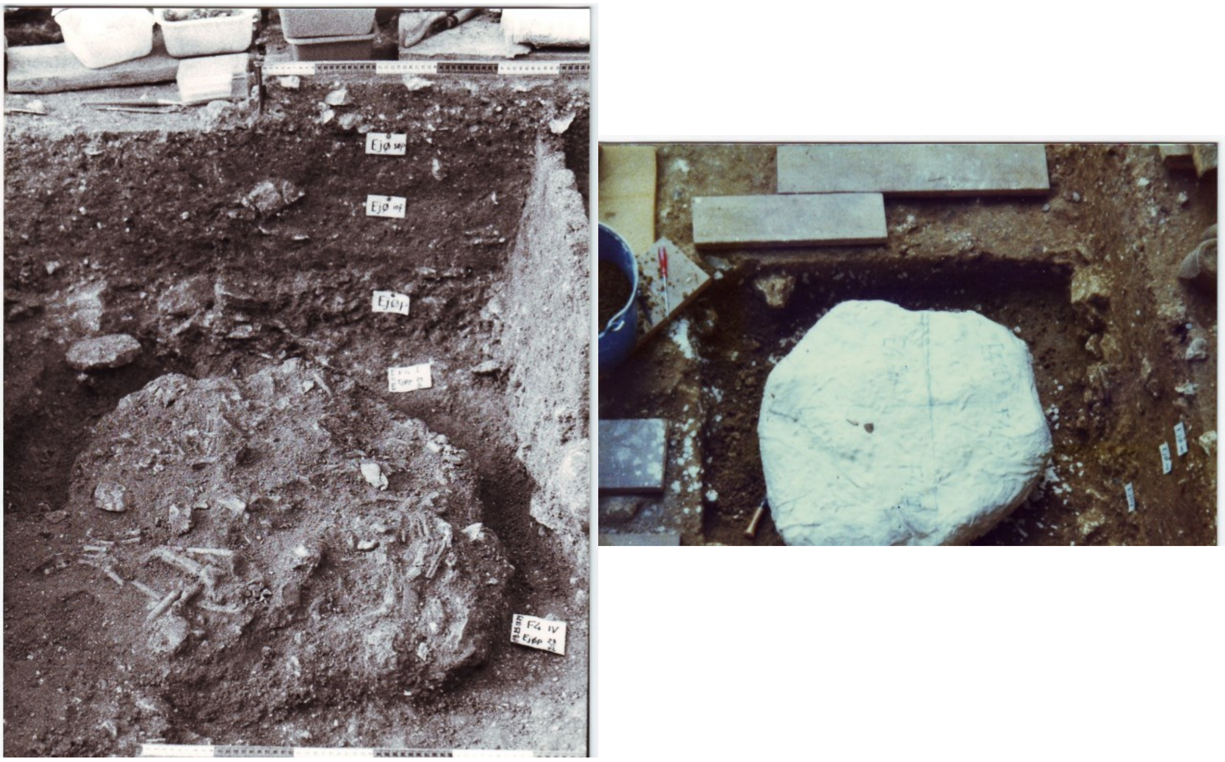

FIGURE S5: Isolation of Neanderthal skeletal material (below left) prior to plastering (below right) and removal for excavation in the laboratory. Photos courtesy of F. Lavaud-Girard.

Techno-typology of diagnostic lithic artefacts (tabl. S1)

All lithic artefacts greater than 2 cm were assigned to the Middle Palaeolithic, Châtelperronian, Indeterminate Upper Palaeolithic based on widely available, well-documented and generally accepted characteristics of these techno-complexes. Apart from clear exceptions (e.g. Châtelperronian points or other retouched tools), this primarily concerned complete flakes, blades and cores. Where doubts persisted, objects were designated 'indeterminate'.

Technology	Criteria (see also refs in Faivre et al., 2017)	References
MIDDLE PALAEOLITHIC		
Levallois	• Levallois cores and typical products (e.g. relatively thin flakes bearing complex or multiple uni- or bipolar scar patterns with or without facetted striking platforms)<br>• Hard-hammer percussion	Boëda 1996, 1994 ; Boëda et al., 1990 ; Dibble and Bar-Yosef (eds) 1995
Discoid	• pseudo-Levallois points<br>• *éclats débordants*<br>• bipyramidal and unifacial cores with secant removals and centripetal, tangential ('cordales') or débordant scar negatives<br>• hard-hammer percussion	Boëda 1993; Locht and Swinnen 1995 ; Peresani (ed.) 2003.
Bifacial	• bifaces<br>• bifacial-thinning flakes	Newcomer 1971 ; Turq, Böeda 1996; Soressi 2002 ; Claud 2008
Laminar Mousterian	• 'elongated' unipolar or bipolar flakes detached by direct hard-hammer percussion	Boëda 1998 ; Revillon 1995 ; Delagnes 2000 ; Soressi 2002
Indeterminate Middle Palaeolithic	• typical Mousterian retouched tools (i.e. side scrapers, notches, denticulates) on Middle Palaeolithic or indeterminate **flake** blanks<br>• flakes with heavily facetted platforms<br>• hard-hammer percussion<br>• centripetal flakes and flake cores<br>• Kombewa *sensu lato* flakes<br>• Truncated-facetted (Nahr Ibrahim or Kostenki)	Bordes 1961 ; Tixier and Turq 1999 ; Dibble and McPherron 2007
UPPER PALAEOLITHIC		
Châtelperronian	• small blades (detached by soft stone hammer percussion)<br>• Châtelperronian points<br>• backed blades and bladelets<br>• semi-circular end scrapers<br>• Châtelperronian blade cores	Pelegrin, 1995 ; Connet, 2002 ; Bachellerie 2011 ; Roussel 2013 ; Roussel et al. 2016 ; Bodu et al. in press

TABLE S1: Techno-typological criteria.

Tool counts, surface alterations, and artefact photos (tabl. S2-5, fig. S6-16)

Blank Type	Retouched > 4 cm	Unretouched > 4cm	Retouched > 4 cm	Unretouched > 4cm	Tools and Pseudo Tools	TOTAL
Indeterminate Middle Paleolithic	244	755	0	0	51	1050
Pseudo-Levallois Point	26	36	0	0	9	71
Centripetal Flakes	70	39	0	0	14	123
Eclat débordant	36	25	0	0	18	79
Blade	0	0	3	0	4	7
Blade Fragment	0	0	3	39	38	80
Bladelet	0	0	0	3	0	3
**TOTAL**	**376**	**855**	**6**	**42**	**134**	**1413**

TABLE S2: Middle and Upper Palaeolithic components of the reattributed EJOP sample by blank type

	Middle Paleolithic	Upper Paleolithic	Non-Attributed	TOTAL
Cores	56	2 (Châtelperronian)	39	97
Non-retouched lithics	1231	48 (3 bladelets)	2988	4267
Tools	35	41	38	114
Pseudo Tools	58	0	19	77
**TOTALS**	**1380**	**91 (88 Châtelperronian)**	**3084**	**4555**

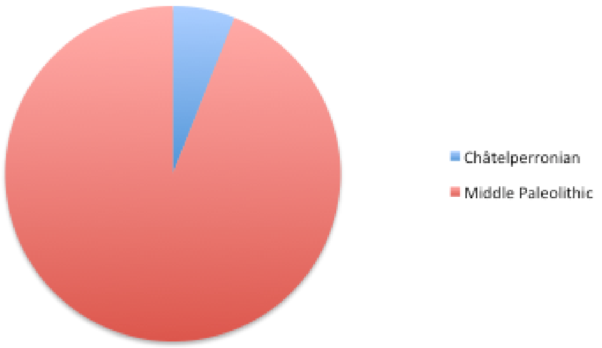

TABLE S3: Overall composition of the reattributed EJOP sample – 94% Middle Palaeolithic (n=1380), 6% Châtelperronian (n=88)

> 4 cm				
	Type 1	Type 2	Type 3	n=
**Middle Paleolithic**	35.69%	45.35%	18.97%	539
**Châtelperronian**	68%	32%	0	25
**Indeterminate**	29.61%	60.85%	9.53%	493

< 4 cm				
	Type 1	Type 2	Type 3	n=
**Middle Paleolithic**	69.37%	24.55%	6.08%	841
**Châtelperronian**	57.97%	37.68%	4.35%	66
**Indeterminate**	75.07%	22.96%	1.97%	2591

TABLE S4: Surface alterations of lithic objects by chrono-cultural attribution of reattributed EJOP sup sample (n=4555). Type 1 = Fresh, Type 2 = some edge modification, Type 3 = rolled and lustred.

Partially cortical flake	3
Centripetal flake	1
Eclat débordant	2
Fragments greater than 25 mm	13
Fragments less than 25 mm	47
**TOTAL**	**66**

**Middle Palaeolithic**	**5**
**Châtelperronian**	**0**
**Upper Palaeolithic**	**0**

TABLE S5: Lithic material recovered from the plastered block containing the Neanderthal skeleton.

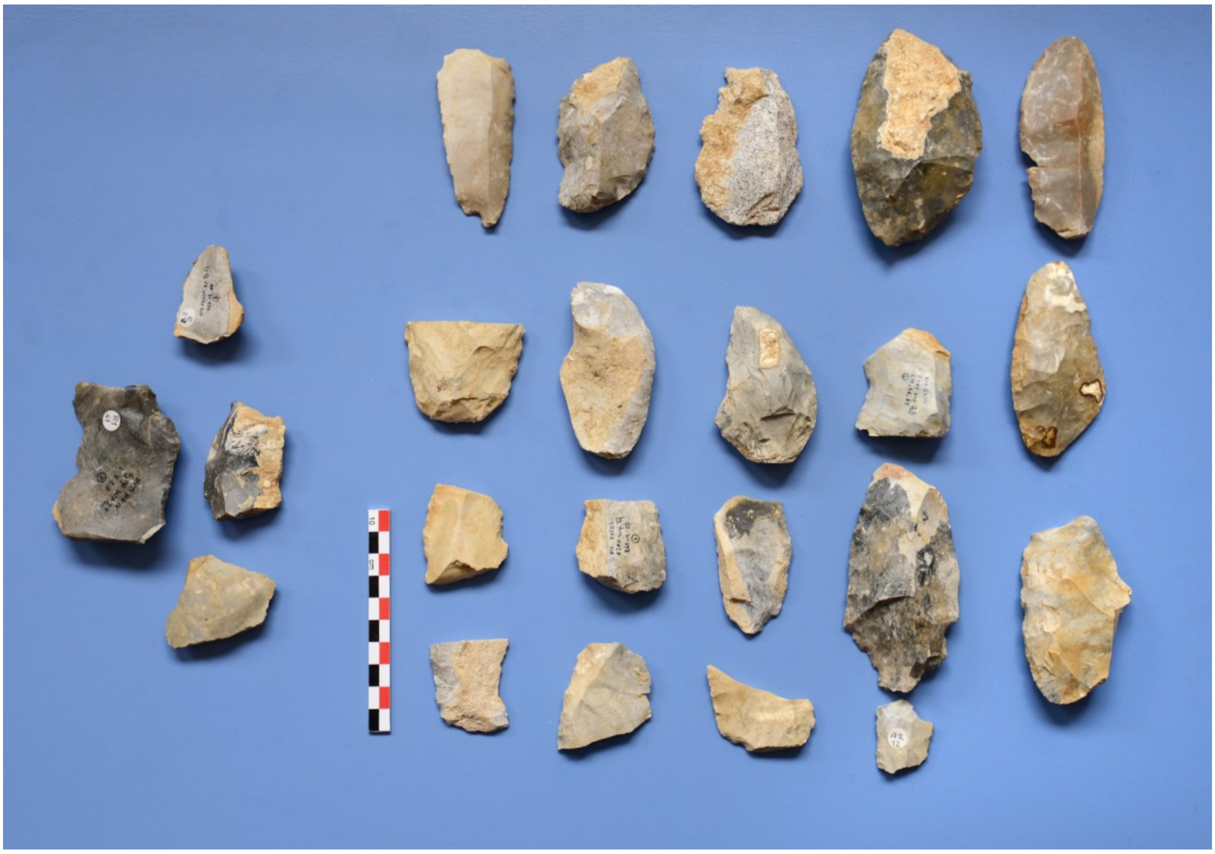

FIGURE S6: Middle Palaeolithic scrapers from the reattributed EJOP sup assemblage

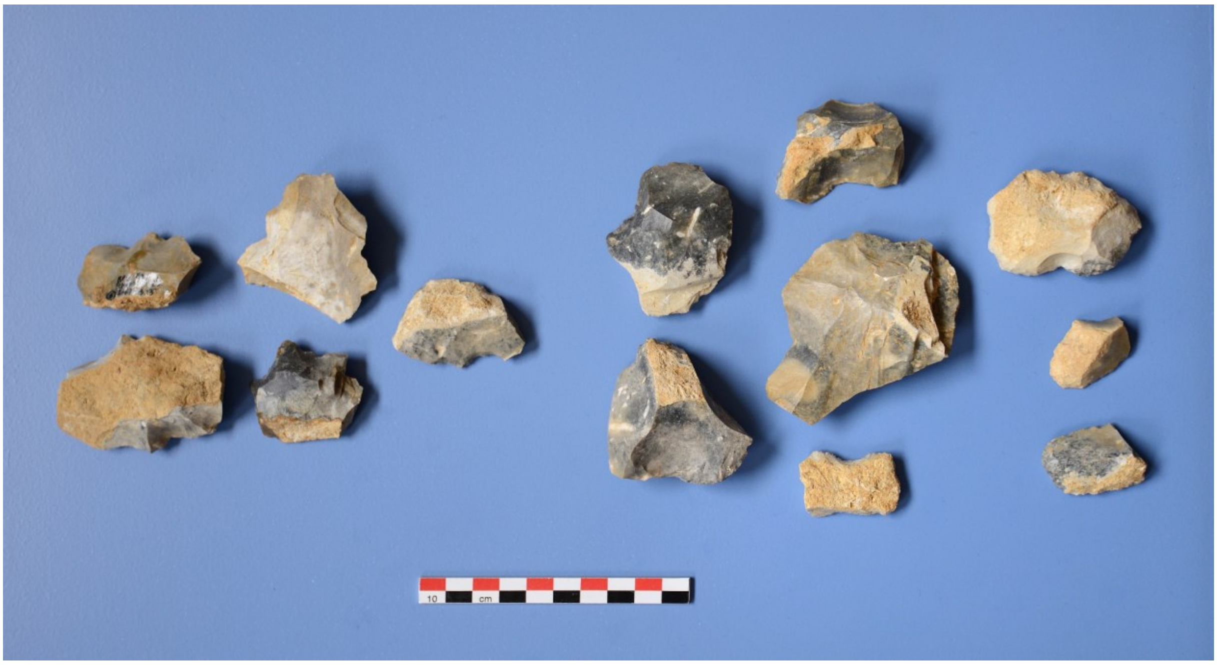

FIGURE S7: Notches and denticulates from the reattributed EJOP sup assemblage

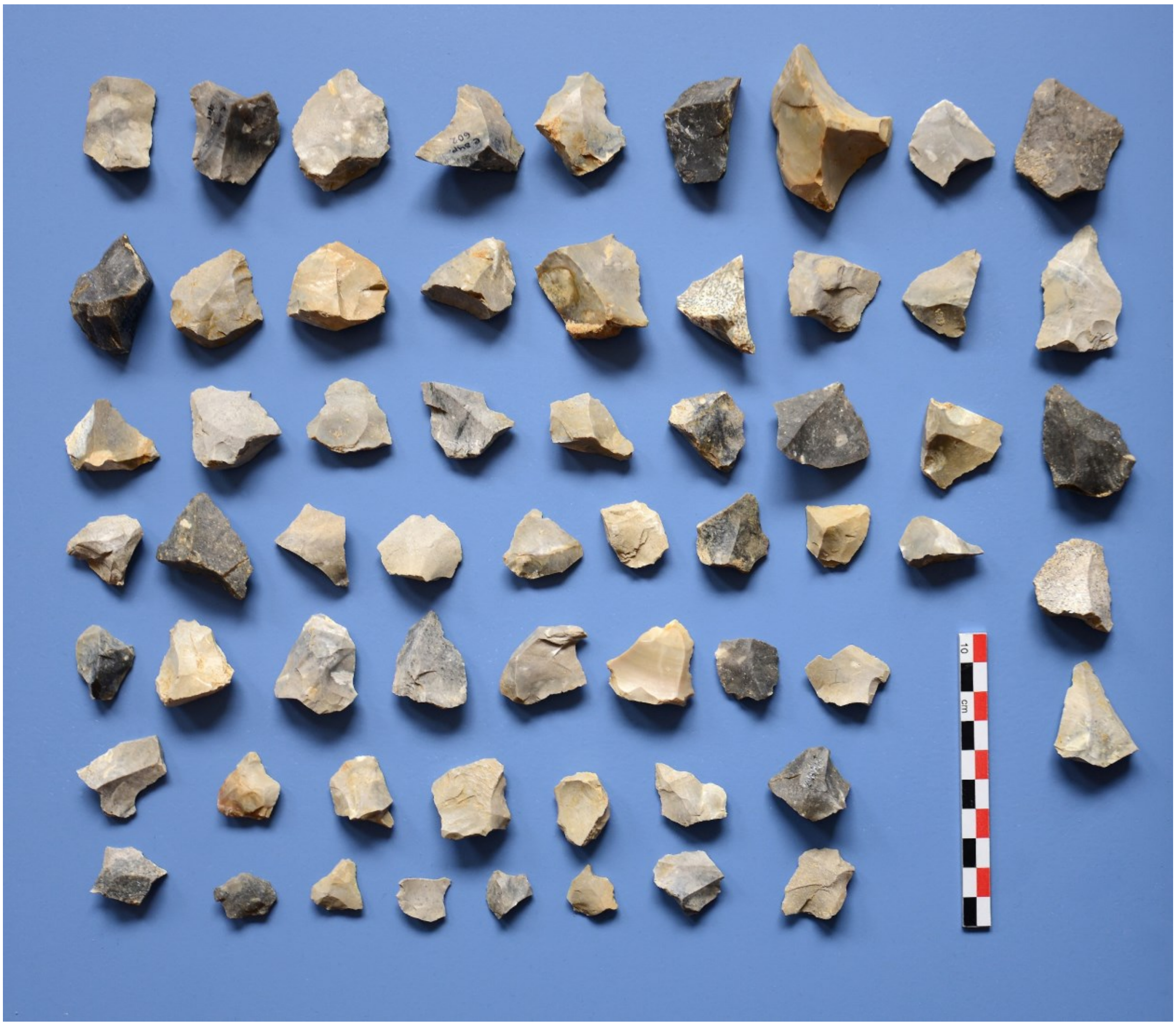

FIGURE S8: Pseudo-Levallois points from the reattributed EJOP sup assemblage

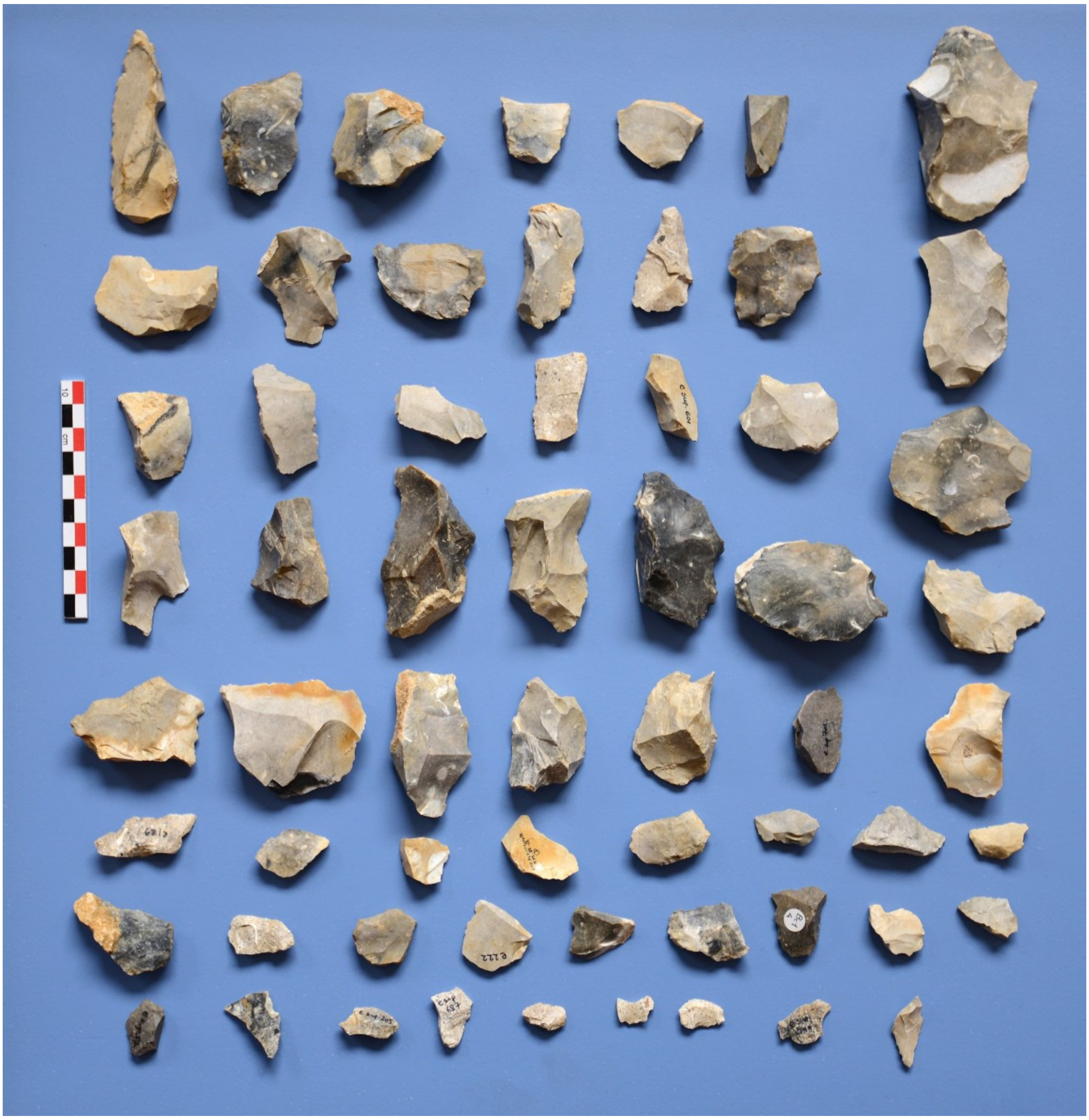

FIGURE S9: Eclats débordants from the reattributed EJOP sup assemblage

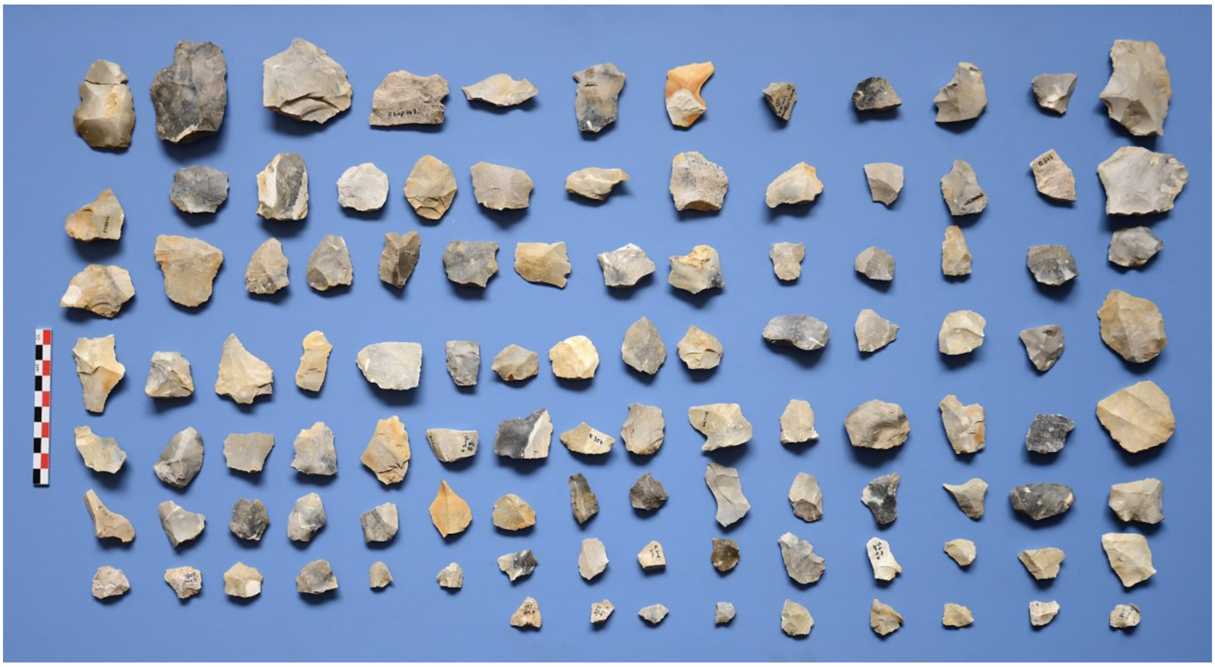

FIGURE S10: Centripetal flakes (including Levallois products) from the reattributed EJOP sup sample assemblage

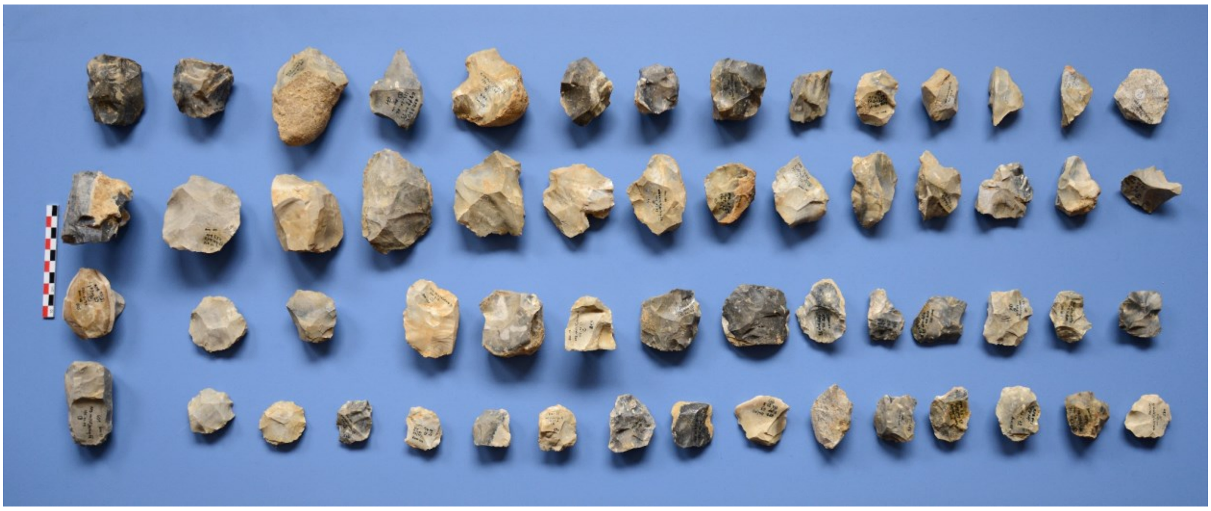

FIGURE S11: Middle Palaeolithic cores from the reattributed EJOP sup sample assemblage

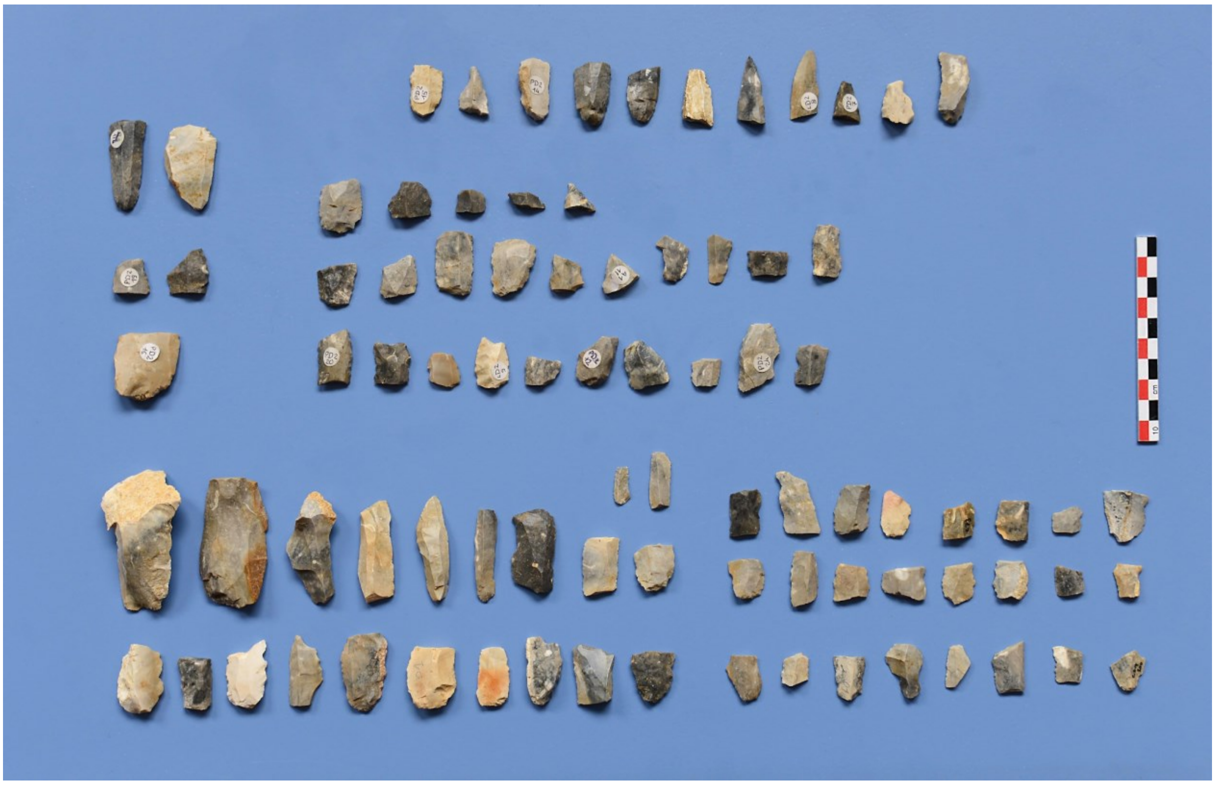

FIGURE S12: Châtelperronian component from the reattributed EJOP sup assemblage

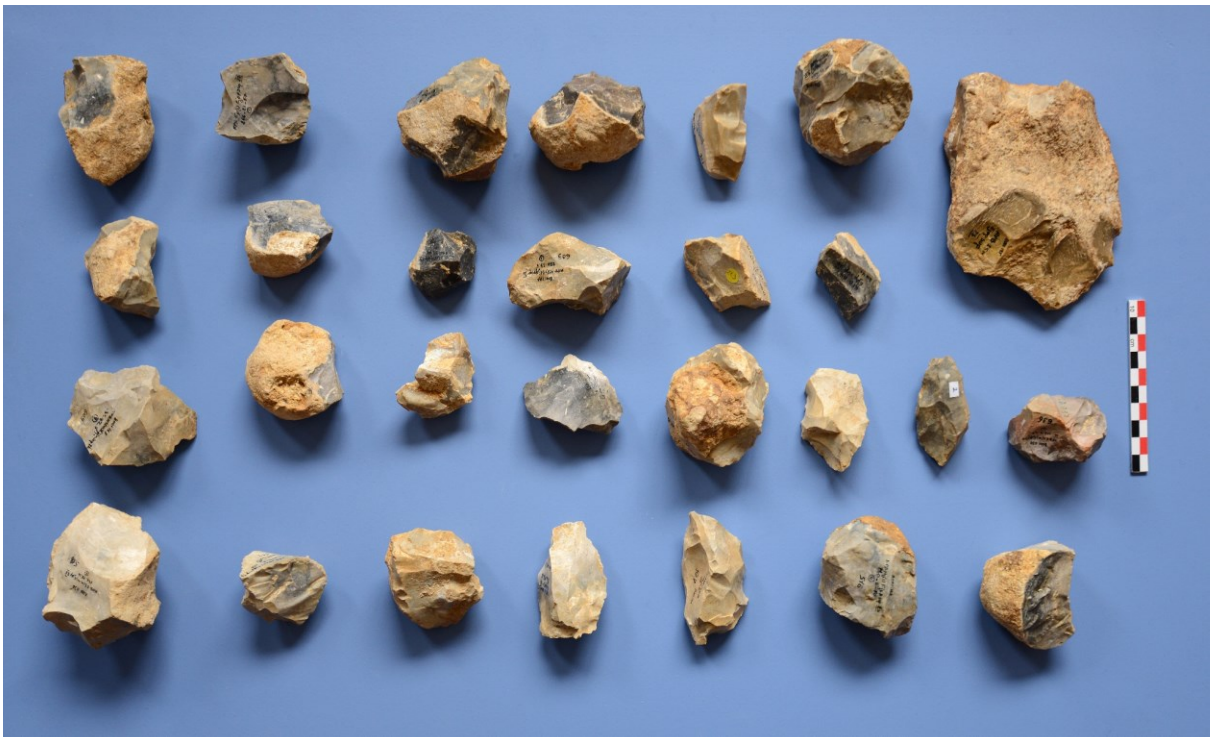

FIGURE S13: Indeterminate cores from the reattributed EJOP sup assemblage

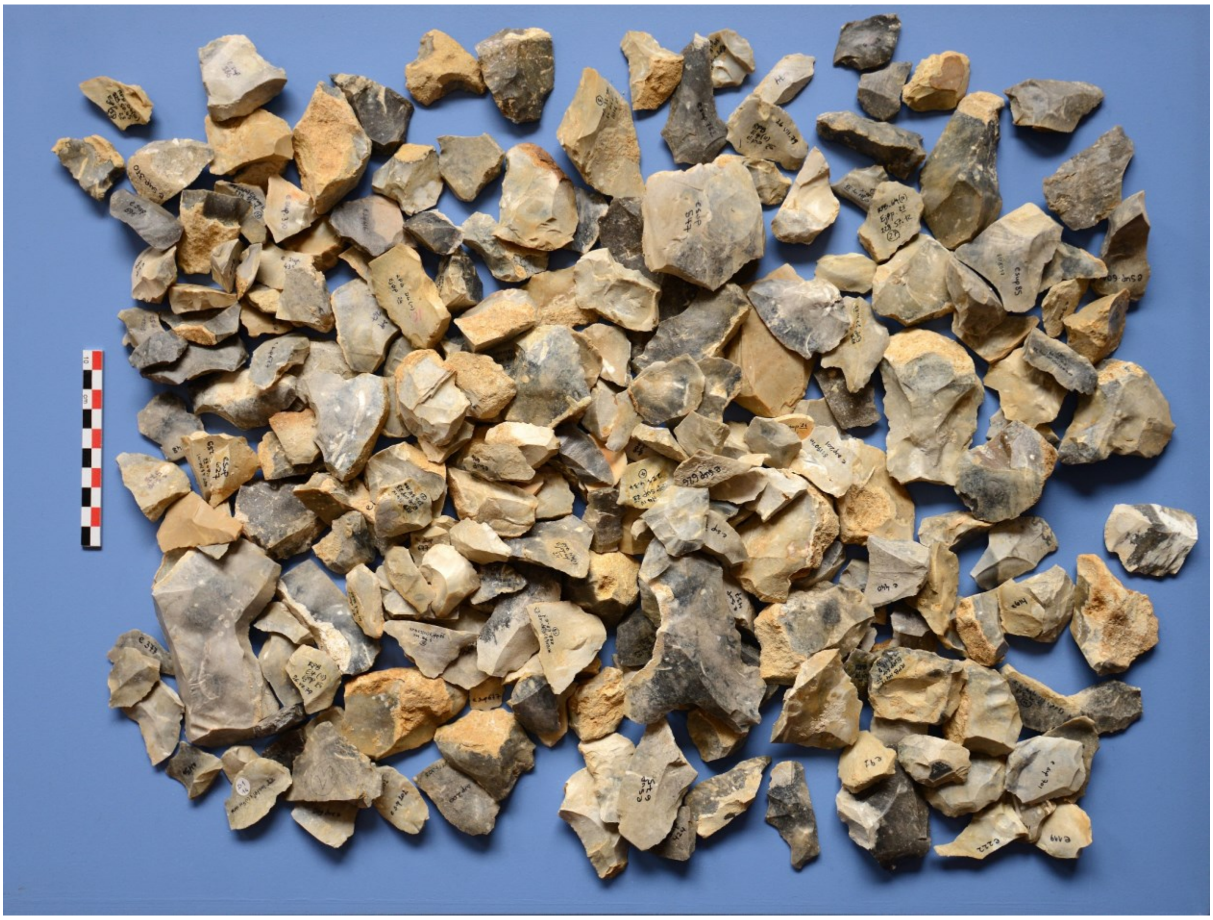

FIGURE S14: Indeterminate lithic objects *greater than 4cm* from the reattributed EJOP sup sample (n=4555)

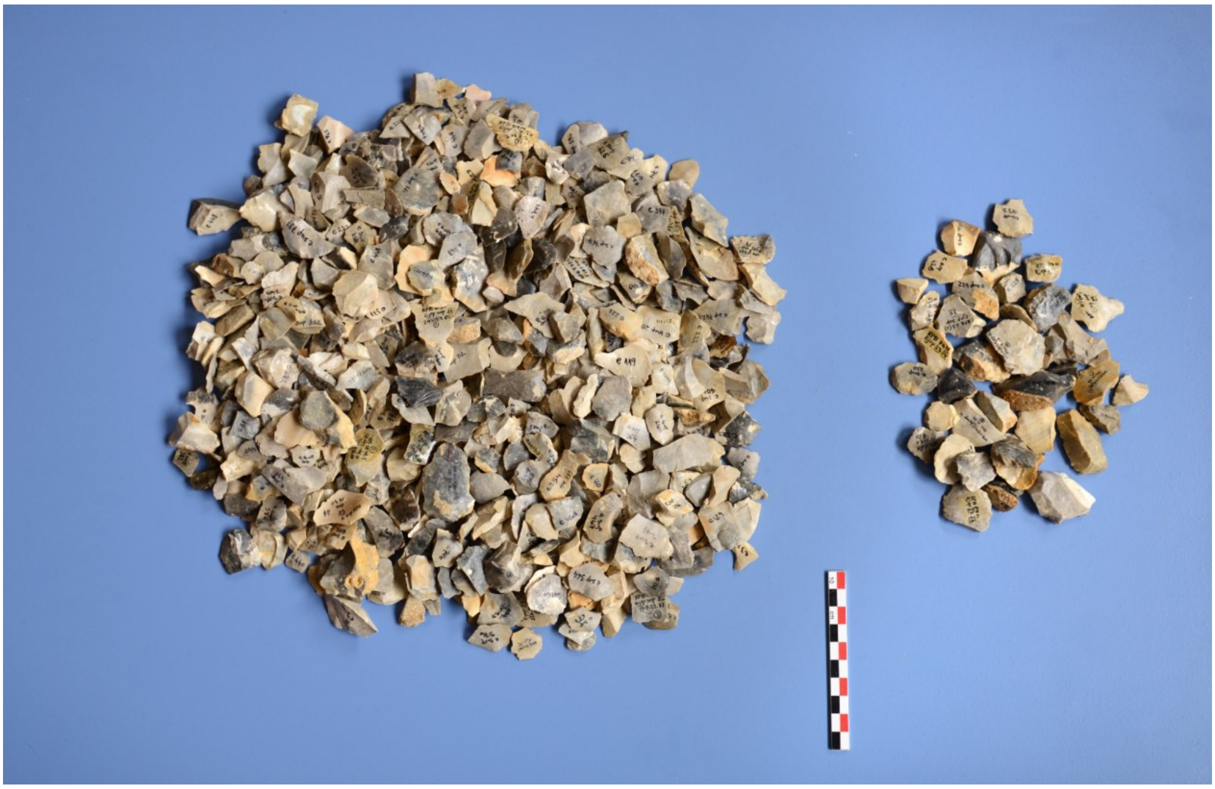

FIGURE S15: Indeterminate lithic objects *less than 4cm* from the reattributed EJOP sup assemblage

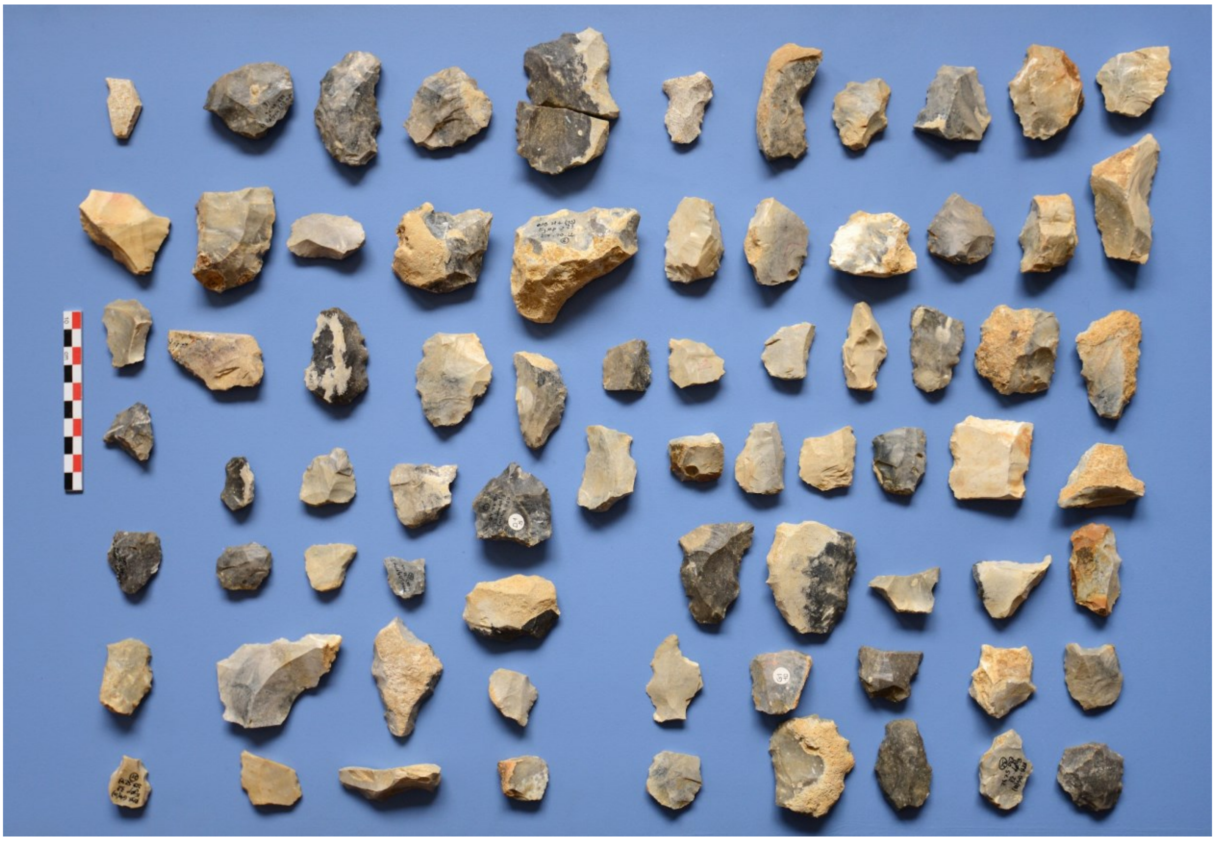

FIGURE S16: Pseudo-tools from the reattributed EJOP sup assemblage

Analytical protocol for the evaluation of surface alterations using confocal microscopy (A. Galland, fig. S17-18)

In order to assess variability in surface alterations within the immediate environment of the site we built two reference samples from locally available raw materials (Upper Cretaceous flint) identical to those present in EJOP sup

- The geological sample consisted of Coniacian flint from alterites and colluvial deposits in the plateau above the site, and Santonian flint collected from colluvial deposits 3km west of the site.
- The experimental sample comprised flakes detached from these different geological samples using direct hard-hammer percussion. These flakes were not altered by post-depositional surface modifications.

The data were then compared to the archaeological sample from EJOP sup, which consisted of 5 Châtelperron points from different areas of the site and 8 flakes from a 2 cm spit of a 25 sq. centimetre sub-square (square F5 – sub-square III) in the immediate vicinity of the Neanderthal remains.

The microtopography measurements and 3D scans (Fig S17), representing a surface of 877x666 µm were realised with a Sensofar S neox confocal microscope (Sensofar, Barcelona). Surfaces were scanned with a 20x lens (0.45 NA) allowing a lateral sampling interval of 0.645 µm, a vertical resolution of 8 nm, and an optical resolution of 0.31µm. Prior to measurements, the surfaces were cleaned with alcohol (90% modified) in order to remove any residues connected to the handling of the material. The surfaces were then analysed using SensoMap 7.2 software (Sensofar, Barcelona). First, we used automatic levelling correction obtained through the Least Squares Plane Method then outliers due to measurement errors were removed and filled with non-measured points. Finally, we separated the wavelengths corresponding to roughness from those related to surface form and waviness using a Gaussian filter with a 0.25mm cut-off. The parameters extracted from the roughness area were taken from the ISO norm 25178, with each corresponding to a specific statistical measurement of an area. For all samples, 5 to 10 measurements were recorded for each scar negative. The outlying roughness values were removed using boxplots. In all, 649 measurements for the frame of reference and 256 measurements for the archaeological sample were obtained. We chose to use the two parameters, square root mean height (Sq in µm) and developed surface ratio (Sdr expressed as a percentage), that best characterized microtopography and irregularities of the surface, respectively.

The results show that the alterations of the archaeological sample to be highly heterogeneous and overlap with the variability of alterations from the local environment (Fig. S18).

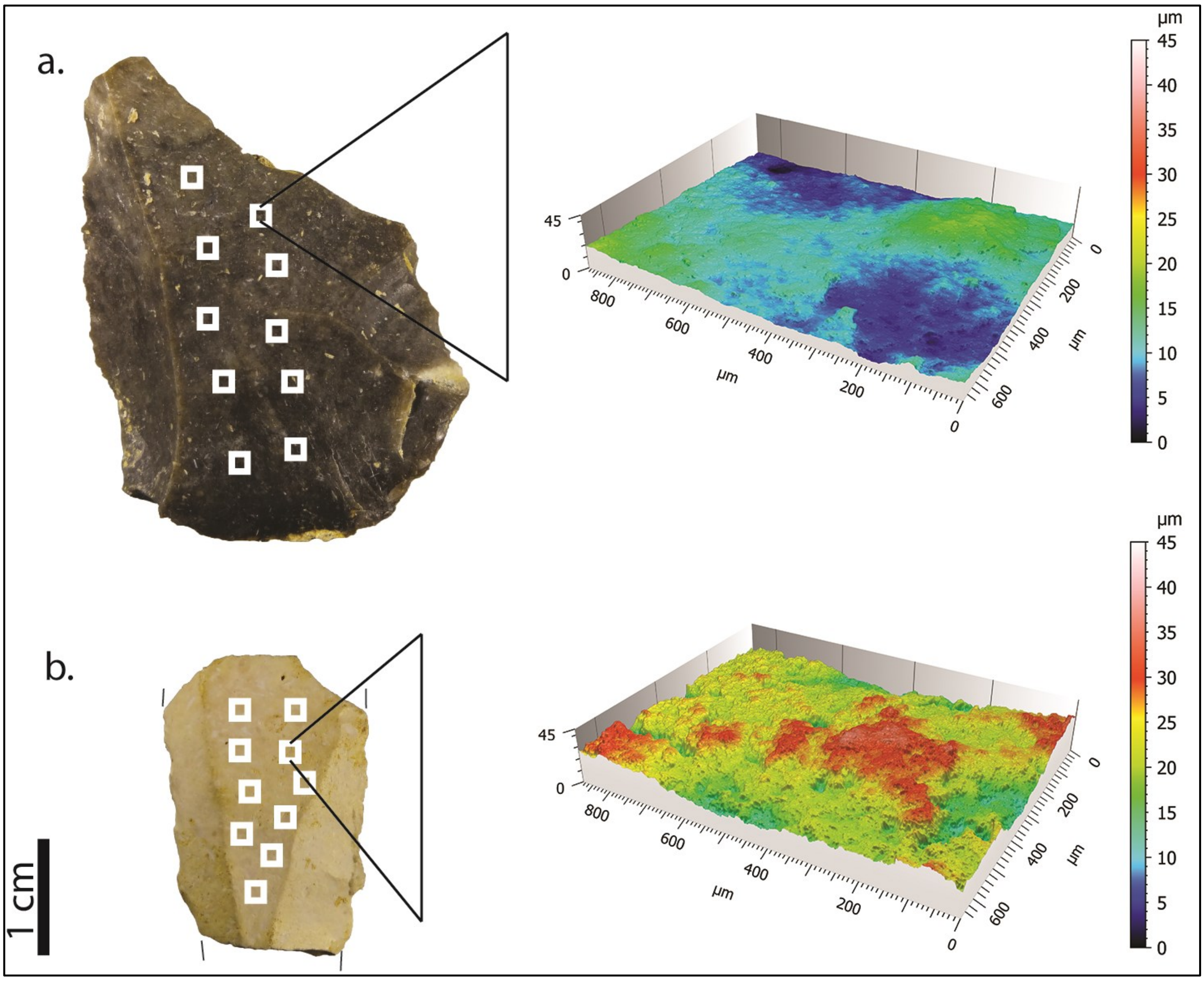

FIGURE S17: 3D scans obtained with confocal microscopy. Two artefacts from EJOP sup: a. macroscopically lustred surface, b. mesial fragment of Châtelperron point with a macroscopically patinated surface. Note the difference in surface topography.

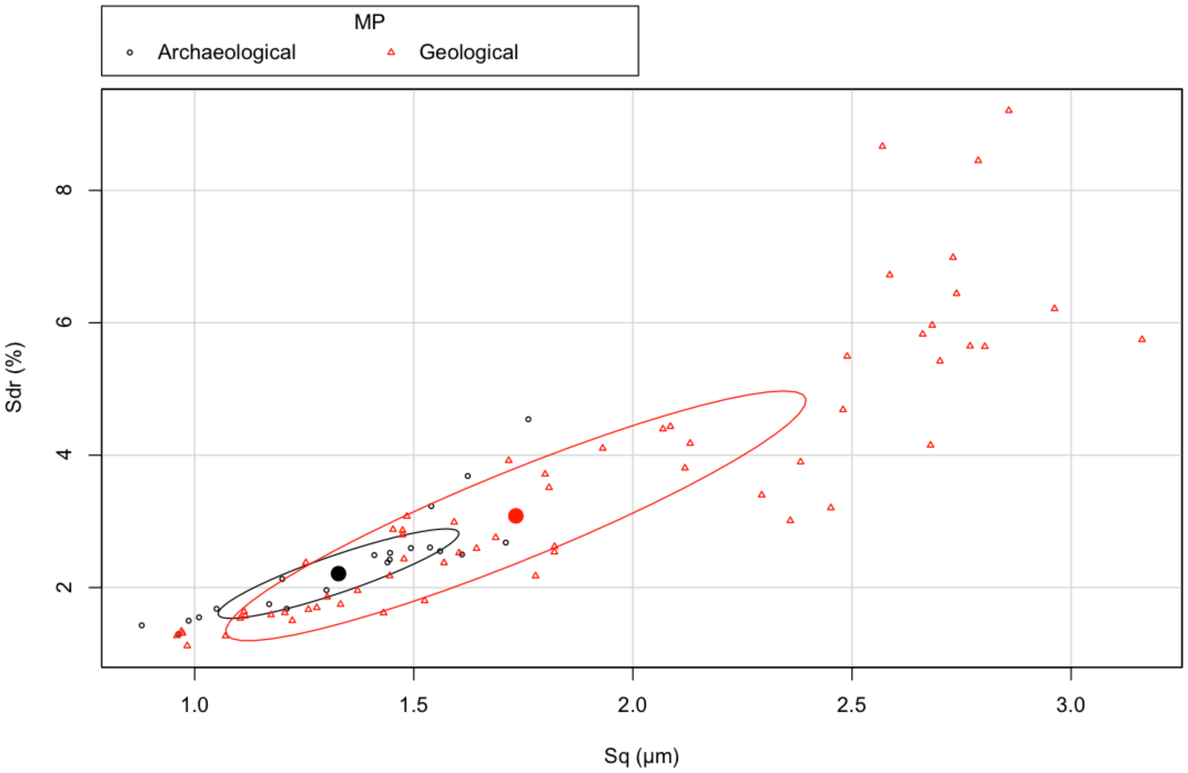

FIGURE S18: Measurements obtained for the geological sample (red) and the archaeological sample (black). The ellipses represent 50% of the variance.

Systematic testing for conjoinable fragments (tabl. S6-S7, fig. S19-20)

This method consists of systematically testing for 'break conjoins' (Tixier, 1978) between all blade and flake fragments in a given assemblage (Bordes 1998, 2000). This method has a number of advantages compared to 'debitage' conjoins: first, 'break' conjoins are far simpler to find as the entirety of the two surfaces of the break are likely to be conjoinable in the majority of cases and, second, this method allows for a quantification of the success rate based on a calculable number of potential conjoins, and three, if properly organised and systematically employed, all possible connections between pieces can be tested.

In the case of Saint Césaire, all identifiable fragments from all stratigraphic subdivisions of Lévêque's EJOP (i.e. EJOP inf, EJOP sup, and undifferentiated) with a break longer than 15 mm (n=1441) were systematically tested for conjoins. This size limit assures that each possible conjoin would likely have been collected during Lévêque's excavations. In order to ensure maximum reliability, all lithic artefacts, including non-piece plotted material, were included from the entirety of the excavated surface of Lévêque's EJOP (32 sq. m). The material was separated by break type (proximal, mesial, distal) and laid out on a table according to raw material, presence and lateralisation of cortex, break size and type (Fig. S19). Each piece was than systematically compared; proximal to distal and mesial, mesial to mesial, and mesial to distal (See Table S6 for counts of each fragment type).

	Non-cortical	Cortical	Total
**Distal fragments**	316	308	624
**Mesial fragments**	138	71	209
**Proximal fragments**	364	244	608
**Total**	818	623	1441

TABLE S6: La Roche à Pierrot, EJOP, Number by type of fragments included in the systematic testing program.

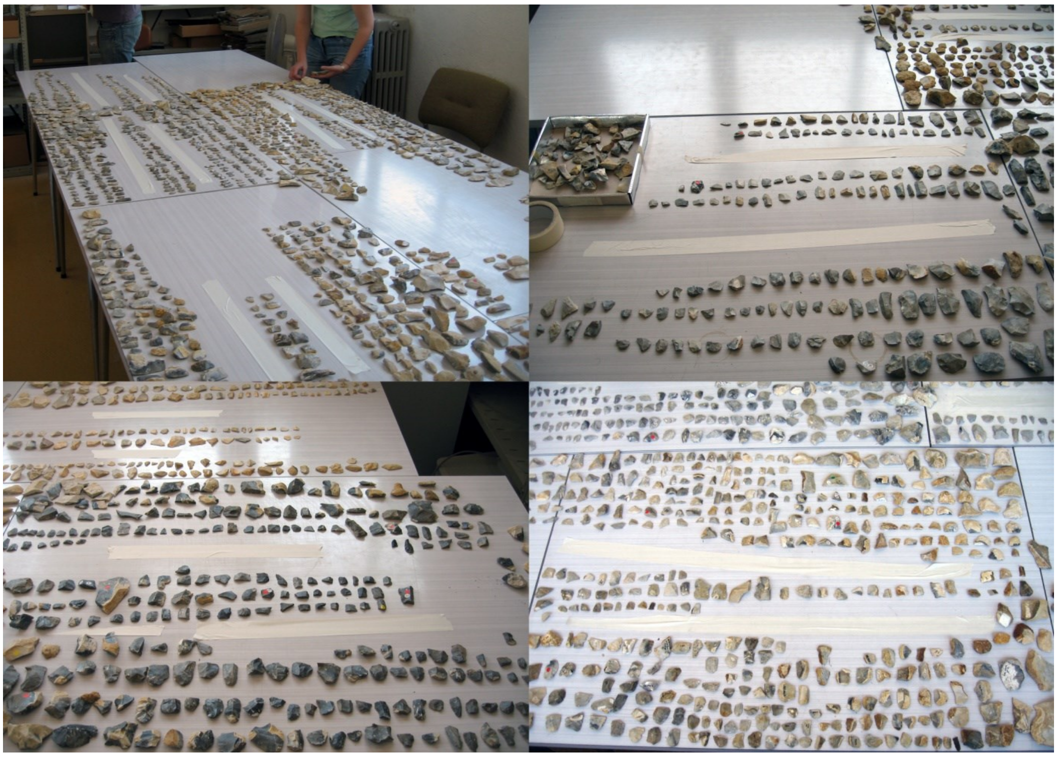

FIGURE S19: Flake and blade fragments by portion from EJOP during systematic testing for conjoins

Only 29 connections (see Table S7 for their distribution) were found out of a total of 680,352 possible connections, for a success of rate of 4.02 % and a refitting index of $4.262*10^{-5}$.(Bordes 1998)

	EJOP	EJOP sup	EJOP inf
**EJOP**	12	7	1
**EJOP sup**		7	0
**EJOP inf**			2

TABLE S7: La Roche-à-Pierrot, EJOP, number of conjoins by assemblage attribution

The percentage of conjoins (P) and the refitting index (I) were calculated as:

$$\mathbf{P}= \frac{number of connections found}{number of pieces considered} X100$$

$$\mathbf{I}= \frac{number of successful connections}{number of connections tested}$$

In this formula, the number of successful comparisons corresponds to the number of conjoins found (in the case of EJOP, n= 29), with the number of overall comparisons representing the number of possible conjoins bearing in mind that the a proximal fragment potentially conjoins with either a mesial or distal fragment and that a mesial fragment can connect with a second mesial fragment or a distal fragment (Fig. 20). Therefore, **A** = the number of proximal fragments, **B** = the number of mesial fragments and **C** = the number of distal fragments. The number of comparisons can thus be calculated as (Bordes, 1998):

Number of connections tested = (A*B) + (B*C) + (B*B-1) + (A*C)

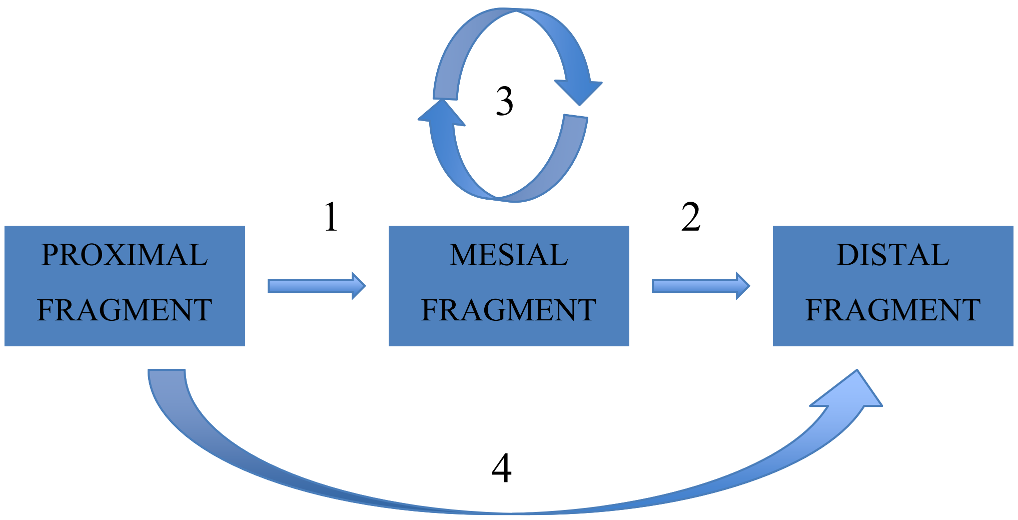

FIGURE S20: Potential connections (modified after Bordes, 1998).

Comparative data for systematic testing of break conjoins (tabl. S8)

Site	Success Rate	Interpretation	Reference
Le Piage	12.3	Inter-level mixing but overall cultural sequence generally preserved	Bordes 2000
Caminade	8.1 to 18.2	Inter-level mixing but overall cultural sequence generally preserved	Bordes 2002
Roc-de-Combe	2 to 4	Inter-level mixing but overall cultural sequence generally preserved. However, small excavated surface	Bordes 2002
Corbiac-Vignoble II	15	Summit of the level partially disturbed by cryoturbation / very good preservation	Bordes and Tixier 2006
Canaule II	35	Exceptionally well preserved site / high export of blanks	Bachellerie *et al.* 2008

TABLE S8: Comparative data for the systematic testing of break conjoins from Upper Palaeolithic sites mentioned in the text.

Lithic raw materials (S. Caux, fig. S21)

Two different groups of siliceous raw materials were exploited at Saint Césaire and reflect two different provisioning strategies.

Group 1: non-local flint

- « Grain de mil » flint that outcrops approximately 10 km east of the Site. This black or blond flint is characterised by numerous Bryozaires fragments accompanied by benthic foraminifera (Caux 2015 ; Caux and Bordes 2016))
- Middle Turonian flint from Ecoyeux, available 7 km north of the site. This blond to bluish, more or less translucent flint is poor in inclusions (Soressi 2010; Caux 2015).

Group 2: flint available in the immediate vicinity of the site

- Black Middle Santonian flint poor in inclusions but characterised by large, complete Bryozaires
- Grey Lower Santonian flint formed from sponges whose skeleton is still evident in the often poorly silicified centre.
- Grey to black Coniacian flint present as highly variable blocks, both in terms of size and uniformity, containing limited quantities of sub-complete Bryozoaires.

The diversity of the first group of raw materials can be explained by the local geomorphological context. The rock shelter is located at the base of a Turonian cliff directly overlain by, respectively, Coniacian (Fig. S21, n°3) and Santonian (Fig. S21, n°2) limestone formations followed by Middle and Upper Santonian alterites (Fig. S21, n° 1) containing residualised siliceous deposits. Different Coniacian and Santonian flints would have been immediately available to prehistoric groups in the alluvial and colluvial deposits of Coran at the base of the cliff. This would equally explain the presence of numerous geofacts in EJOP sup that would have been transported by gravitational processes along the slope, as evident by the numerous shocks and 'natural' removals suffered by this material. Some of the better quality blocks of these locally available raw materials (grey Lower Santonian and Coniacian flint) were exploited by the inhabitants. This local provisioning strategy was accompanied by the use of better quality Group 1 flints.

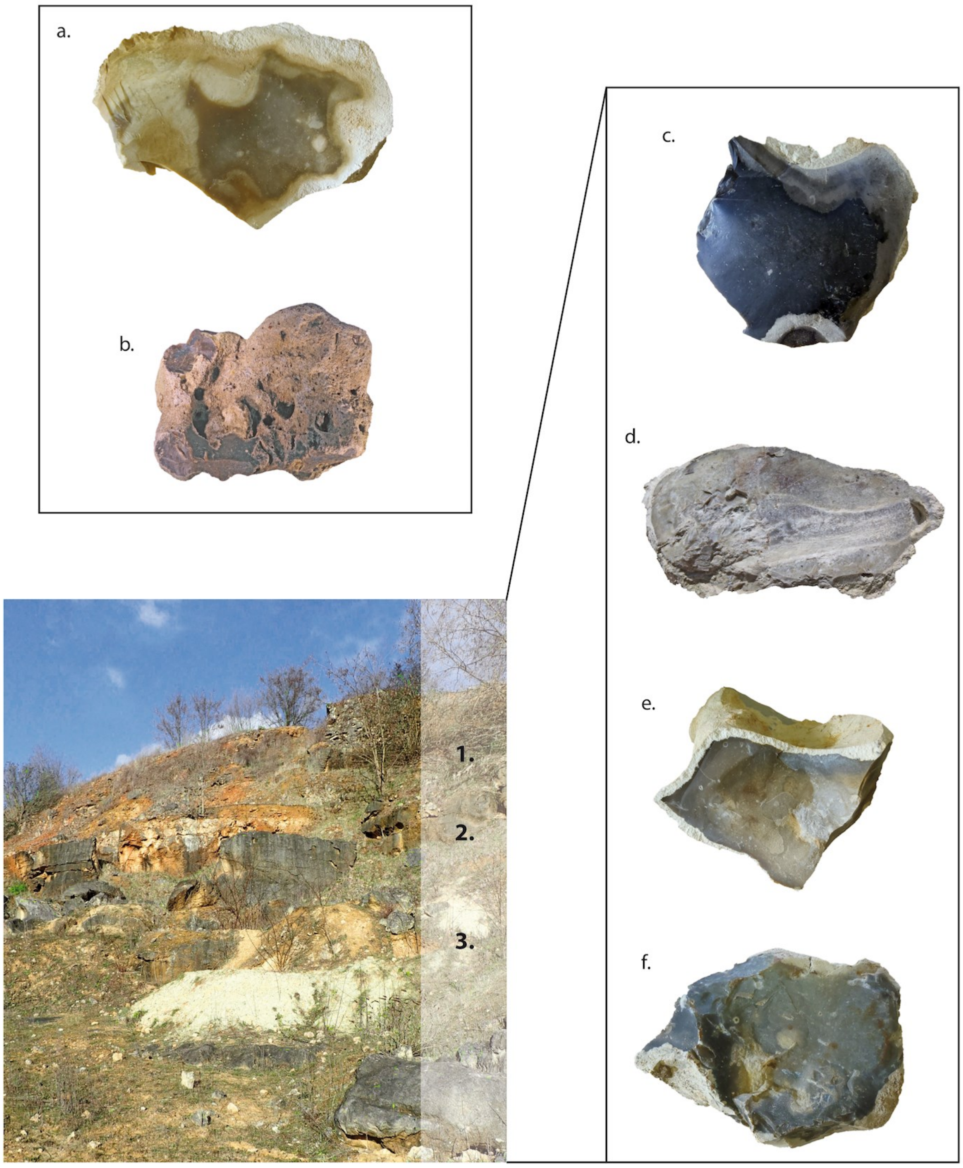

FIGURE S21. Macroscopic traits of flint used at Saint Césaire.

Local flint (10 to 20 km): a. Turonian flint from Ecoyeaux et b. Grain de Mil flint from Saintes

Raw materials available in the alterites on the plateau immediately overlying the site: c. black Middle Santonian flint, d. grey Lower Santonian flint with fossil sponges, e and f. grey to black Coniacian flint.

Outcrops on the slopes overlying the site: 1. red Santonian alterites; 2. white to grey Lower Santonian limestone, 3. grey to green Coniacian sands.

Bibliography

Bachellerie F (2011). Quelle unité pour le Châtelperronien? Apport de l'analyse taphonomique et techno-économique des industries lithiques de trois gisements aquitains de plein air: le Basté, Bidart (Pyrénées-Atlantiques) et Canaule II (Dordogne). Unpublished Doctoral Dissertation. University of Bordeaux.

Bodu P, Salomon H, Lacarrière J, Baillet M, Ballinger M, Naton HG, Théry-Parisot I (2017) Un gisement châtelperronien de plein air dans le Bassin parisien : Les Bossats à Ormesson(Seine-et-Marne). Gallia Préhistoire 57: 3-64

Boëda E (1993) Le débitage discoïde et le débitage Levallois récurrent centripède. *Bull la Société préhistorique française*:392–404.

Boëda E (1994) Le concept Levallois: variabilité des méthodes. *Monogr du CRA* 9:280.

Bordes F (1961) *Typologie du Paléolithique ancien et moyen* (Bordeaux). Delmas.

Bordes J.-G (1998) L'Aurignacien 0 en Périgord : analyse des données. Un exemple d'application d'une méthode de quantification des remontages d'intéret stratigraphique : Caminade est, couche G. Unpublished Masters Thesis. Université de Bordeaux.

Bordes J-G (2000) La séquence aurignacienne de Caminade revisitée : l'apport des raccords d'intérêt stratigraphique. The Aurignacian sequence at Caminade Est revisited : contribution of the refittings to stratigraphie study. Paléo 12, 387–407.

Caux S (2015) Du territoire d'approvisionnement au territoire culturel - Pétroarchéologie et techno-économie du silex Grain de mil durant l'Aurignacien dans le Sud-ouest de la France. Thèse de doctorat, Bordeaux: Université de Bordeaux.

Caux S, Bordes J-G (2016) Le silex Grain de mil, ressource clé du Sud-Ouest de la France au Paléolithique : caractérisation pétroarchéologique et clé de détermination en contexte archéologique, Paléo, 27, pp. 105 – 131

Claud É (2008) Le statut fonctionnel des bifaces au Paléolithique moyen récent dans le Sud-Ouest de la France. Étude tracéologique intégrée des outillages des sites de La Graulet, La Conne de Bergerac, Combe Brune 2, Fonseigner et Chez-Pinaud / Jonzac.*Unpublished Doctoral Dissertation* (Université de Bordeaux).

Connet N (2002) Le Châtelperronien, réflexions sur l'unité et l'identité techno-économique de l'industrie lithique :l'apport de l'analyse diachronique des industries lithiques des couches châtelperroniennes de la grotte du Renne à Arcy-sur-Cure (Yonne). Unpublished Doctoral Dissertation, Université de Lille 1.

Delagnes A (2000) Blade Production during the Middle Paleolithic in Northwestern Europe. Acta Anthropologica Sinica (Supplement to Vol. 19), pp.169-176.

Dibble H, Bar-Yosef O (1995). The Definition and Interpretation of Levallois Technology (No. 23). Prehistory Press.

Dibble H, McPherron SP (2007) Truncated-Faceted Pieces: Hafting Modification, Retouch, or Cores? *Tools versus Cores Alternative Approaches to Stone Tool Analysis*, pp 198–222.

Faivre J-P, Gravina B, Bourguignon L, Discamps E, Turq A (2016) Late Middle Palaeolithic lithic technocomplexes (MIS 5–3) in the northeastern Aquitaine Basin: Advances and challenges. *Quat Int*. doi:10.1016/j.quaint.2016.02.060.

Levêque F (1997) Le Passage du Paléolithique moyen au Paléolithique supérieur: Données stratigraphiques de quelques gisements sous-grotte du sud-ouest. Quaternaire 8, 279–287.

Locht JL, Swinnen C (1994) Le débitage discoïde du gisement de Beauvais (Oise) : aspects de la chaîne opératoire au travers de quelques remontages. *Paléo* 6(1):89–104.

Newcomer MH (1971) Some quantitative experiments in handaxe manufacture. *World Archaeol* 3(1):85–94.

Pelegrin J (1995). Technologie lithique: le Châtelperronien de Roc-de-Combe (Lot) et de La Côte (Dordogne). Paris, Edition du CNRS, Cahiers du Quaternaire, 20.

Peresani M (2003) *Discoid Lithic Technology: Advances and Implications* (Archaeopress, Oxford). BAR Intern.

Roussel M (2013) Méthodes et rythmes du débitage laminaire au Châtelperronien : comparaison avec le Protoaurignacien. *Comptes Rendus Palevol* 12(4):233–241.

Roussel M, Soressi M, Hublin J-J (2016) The Châtelperronian conundrum: Blade and bladelet lithic technologies from Quinçay, France. *J Hum Evol* 95:13–32.

Soressi M (2002) Le Moustérien de tradition acheuléenne du sud-ouest de la France: Discussion sur la signification du faciès à partir de l'étude comparée de quatre sites : Pech-de-l'Azé I, Le Moustier, La Rochette et la Grotte XVI. Dissertation (University of Bordeaux).

Soressi M (2010) La Roche-à-Pierrot à Saint-Césaire (Charente-Maritime). Nouvelles données sur l'industrie lithique du Châtelperronien. In: Buisson-Cattil, J., Primault, J. (Eds.): Préhistoire entre Vienne et Charente. Hommes et sociétés du Paléolithique. 25 ans d'archéologie préhistorique en Poitou-Charentes. Association des Publications Chauvinoises, Chauvigny, pp. 191-202.

Thiébaut C, Meignen L, Lévêque F (2009). Les dernières occupations moustériennes de Saint-Césaire : Diversité des techniques utilisées et comportements économiques pratiqués (Charente-Maritime, France). *Bulletin de la Société Préhistorique Française* 106: 691-714.

Tixier J (1978) Méthode pour l'étude des outillages lithiques. Unpublished Doctoral Dissertation. Université de Paris X.

Tixier J, Turq A (1999) Kombewa et alii. *Paléo* 11(1):135–143.
